# Modeled Changes in Potential Grassland Productivity and in Grass-Fed Ruminant Livestock Density in Europe over 1961–2010

**DOI:** 10.1371/journal.pone.0127554

**Published:** 2015-05-27

**Authors:** Jinfeng Chang, Nicolas Viovy, Nicolas Vuichard, Philippe Ciais, Matteo Campioli, Katja Klumpp, Raphaël Martin, Adrian Leip, Jean-François Soussana

**Affiliations:** 1 Laboratoire des Sciences du Climat et de l’Environnement, UMR8212, CEA-CNRS-UVSQ, Gif-sur-Yvette, France; 2 Research Group of Plant and Vegetation Ecology, Department of Biology, University of Antwerp, Wilrijk, Belgium; 3 INRA, Grassland Ecosystem Research Unit, UREP, Clermont-Ferrand, France; 4 European Commission, Joint Research Centre (JRC), Institute for Environment and Sustainability, Ispra (VA), Italy

## Abstract

About 25% of European livestock intake is based on permanent and sown grasslands. To fulfill rising demand for animal products, an intensification of livestock production may lead to an increased consumption of crop and compound feeds. In order to preserve an economically and environmentally sustainable agriculture, a more forage based livestock alimentation may be an advantage. However, besides management, grassland productivity is highly vulnerable to climate (i.e., temperature, precipitation, CO_2_ concentration), and spatial information about European grassland productivity in response to climate change is scarce. The process-based vegetation model ORCHIDEE-GM, containing an explicit representation of grassland management (i.e., herbage mowing and grazing), is used here to estimate changes in potential productivity and potential grass-fed ruminant livestock density across European grasslands over the period 1961–2010. Here “potential grass-fed ruminant livestock density” denotes the maximum density of livestock that can be supported by grassland productivity in each 25 km × 25 km grid cell. In reality, livestock density could be higher than potential (e.g., if additional feed is supplied to animals) or lower (e.g., in response to economic factors, pedo-climatic and biotic conditions ignored by the model, or policy decisions that can for instance reduce livestock numbers). When compared to agricultural statistics (Eurostat and FAOstat), ORCHIDEE-GM gave a good reproduction of the regional gradients of annual grassland productivity and ruminant livestock density. The model however tends to systematically overestimate the absolute values of productivity in most regions, suggesting that most grid cells remain below their potential grassland productivity due to possible nutrient and biotic limitations on plant growth. When ORCHIDEE-GM was run for the period 1961–2010 with variable climate and rising CO_2_, an increase of potential annual production (over 3%) per decade was found: 97% of this increase was attributed to the rise in CO_2_, -3% to climate trends and 15% to trends in nitrogen fertilization and deposition. When compared with statistical data, ORCHIDEE-GM captures well the observed phase of climate-driven interannual variability in grassland production well, whereas the magnitude of the interannual variability in modeled productivity is larger than the statistical data. Regional grass-fed livestock numbers can be reproduced by ORCHIDEE-GM based on its simple assumptions and parameterization about productivity being the only limiting factor to define the sustainable number of animals per unit area. Causes for regional model-data misfits are discussed, including uncertainties in farming practices (e.g., nitrogen fertilizer application, and mowing and grazing intensity) and in ruminant diet composition, as well as uncertainties in the statistical data and in model parameter values.

## Introduction

Consumption of animal-product is increasing in the global food diet, and this trend is projected to continue with large increases in demand for both dairy products and ruminant meat—mainly beef but also lamb and goat [1]. In turn, this rising demand for animal products will impact the demand for animal feed (grown as crops and pasture) and is expected to lead to an intensification of livestock production. Currently, the proportion of pasture-based feed is decreasing, mirrored by increased use of crop-based feeds [2]. Yet grass forage still accounts for around 70% of the global dry matter intake of ruminants (world mean in 1993 [3]). For Europe, Leip et al. [4] estimate that herbage intake provides about 25% of livestock’s protein in the feed, while about 60% are from crops and compound feeds. Of the approximately 13 billion hectares of ice-free land on Earth, 3.4 billion hectares (about 25%) are grasslands, an area 2.3 times larger than that used for arable crops ([5], data of 2010).

Improving grassland productivity and the management of livestock systems is especially important if we are to create economically, socially and environmentally sustainable agriculture [6]. The European livestock sector produces 15 and 21% of the global totals of meat, and of milk and dairy products, respectively, amounting to an economic value of $185 billion ([5], data of 2010). Ruminant livestock is largely bred using grassland as a key resource. Grassland provides fresh herbage for grazing and is also mown to create hay and silage that are predominantly used during the winter period. Most European grassland is infrequently (permanent grasslands) or never (rough pastures) re-sown, but these semi-natural grasslands, which are characterized by a moderate to high plant species diversity, have been increasingly fertilized with both inorganic and organic nitrogen over recent decades [7]. Apart from these so-called rough pastures, which are usually located in mountainous areas with steep slopes and limited accessibility, fulfilling the forage requirement of ruminant livestock requires permanent and sown grasslands in Europe to be used (at farm scale) for both grazing and mowing.

Grassland productivity is not only affected by management, but also responds to rising atmospheric CO_2_ concentration [8] and climate change. Elevated CO_2_ concentration has the dual effect of increasing leaf photosynthesis and reducing stomatal conductivity. These effects tend to increase water-use efficiency [9] and often reduce the consumption of soil moisture [8], but decreased conductance could be offset by increased leaf area index (LAI) in the plant transpired water flux to the atmosphere. Elevated CO_2_ effects on photosynthesis are larger with the C3 species that dominate temperate pastures than with C4 species [10, 11]. Temperate C3 grasses show a typical 30% increase in canopy photosynthesis under doubled atmospheric CO_2_ concentration [12, 13]. Water availability plays a major role in the response of grasslands to climate change, with marked declines in productivity under increased soil water deficits—although there are differences in species response [14]. In the future, elevated atmospheric CO_2_ concentration is expected to reduce the sensitivity of grassland productivity to low precipitation in grassland ecosystems [12, 15]. However, trends and variability in temperature and precipitation, as well as nitrogen limitations, will all interact with the effects of elevated CO_2_ to determine actual changes in grassland productivity [8, 16].

Despite the importance of grasslands in sustaining ruminant livestock production, spatially explicit information about European grassland productivity and its response to climate change is scarce. A few studies have attempted to estimate the potential productivity for individual regions [17–19]. Smit et al. [20] presented a European diagnostic map of grassland productivity derived from census statistics across regions in Europe, integrated with MODIS satellite NDVI observations. However, the mechanisms that control productivity were not investigated in that study. Climate change impacts, including the altered frequency and/or severity of extreme events, and nitrogen deposition impacts on grasslands are better understood at the site or small regional level than at larger scales (e.g., [21, 22]; reviewed by Tubiello et al. [23]).

Projections of climate change impacts on grassland productivity are mostly based on local modeling studies that require many local variables, which limits up-scaling to regional or continental scale (e.g., [24]). Model-based impact assessment studies use relationships to simulate grassland productivity that have usually been derived from field experiments (e.g., [25–27]). However, the applicability of these relationships to larger scales and future conditions is highly uncertain [20]. Process-based vegetation models with equations representing biogeochemical and biophysical mechanisms and the combined response to multiple drivers have the advantage of being applicable from plot to continental scale. Process-based models can also be coupled with regional or global climate models to quantify land-atmosphere feedbacks. This type of mechanistic model is increasingly used for global impact studies focusing on agricultural productivity and terrestrial carbon fluxes (e.g. ISI-MIP, http://www.isi-mip.org).

In this study, a process-based land surface model specifically calibrated and parameterized to simulate the carbon cycle of European managed grasslands ORCHIDEE-GM [28–29] is used to address two questions:

1. What is the geographic distribution of potential (maximal) grassland productivity and potential grass-fed livestock density of managed grasslands over the EU27?
2. How did potential grassland productivity evolve during the past 50 years in response to climate change, atmospheric CO_2_ concentration and nitrogen fertilizer supply?

## Material and Methods

### Model description

ORCHIDEE is a process-based ecosystem model built for simulating global carbon, water and energy fluxes [30]. ORCHIDEE-GM is a version of this model that has been developed to explicitly represent grassland management such as mowing and livestock density. Grassland management was developed by implementing the management module from PaSim [28, 31–33], a grassland model developed initially for site applications, into ORCHIDEE. The equations that differentiate ORCHIDEE-GM from the standard version of ORCHIDEE are described by Chang et al. [29], together with a comparison of both model versions at 11 sites equipped with eddy-covariance and biometric measurements. The model assumes the simple principle that farmers will produce the maximum number of animals that can be fed locally by above ground productivity, optimally combining grazing and mowing (local forage production) [28] as well as nitrogen fertilizer application, although the model does not explicitly simulate the nitrogen cycle [29].

### Effects of nitrogen addition on productivity in ORCHIDEE-GM

Application of nitrogen to pasture leads to higher nitrogen concentration in plant leaves and enhanced leaf photosynthetic capacity [34, 35]. In a meta-analysis gathering many nitrogen addition studies, Xia & Wan [36] reported a 46.5% increase in grass biomass under nitrogen addition, close to another estimate (53% increase of aboveground net primary production (ANPP); [37]). In addition, the effect of N addition to productivity depends on some other factors such as original soil nitrogen availability, nitrogen-fixing plants in grassland community, and management intensity (e.g., herbivores can modify soil N cycling; see a review by Bardgett and Wardle [38]). But the data about these factors are not available over European grassland. Ideally, the relationships among photosynthesis (productivity), leaf nitrogen content, and soil nitrogen availability (can be altered by nitrogen addition) can be modeled with coupled nitrogen/carbon cycling (e.g., O-CN [39]). However, the nitrogen cycle, and thus the interactions between nitrogen and carbon cycles, is not included in ORCHIDEE-GM.

In this study, we tried to apply a simplified function to reproduce empirically the effect of nitrogen addition on grass growth, based on the following fact: i) generally, increased soil nitrogen availability for increased photosynthesis is the result of nitrogen addition, ii) the experimental phenomena [40] showed that grassland productivity was increased along a nitrogen addition gradient, but with a limit after which the productivity will not increase. A variable, representing nitrogen response *N*_*add*_ was added to ORCHIDEE-GM. *N*_*add*_ has a directly additive impact on two photosynthetic parameters, *Vc*_*max*_ *opt* (the maximum rate of Rubisco carboxylase activity), and *J*_*max*_ *opt* (the maximum rate of photosynthetic electron transport), as given by:

$$Vc_{\max}opt=(1+N_{add})\times Vc_{\max}opt^{*}$$ (1)

$$J_{\max}opt=(1+N_{add})\times J_{\max}opt^{*}$$ (2)

where *Vc*_*max*_ opt* and J_*max*_ opt* are the parameter values determining the photosynthetic rates and indirectly, plant productivity and growth in ORCHIDEE. *N*_*add*_ is assumed to be a function of the amount of nitrogen added (*N*_*amount*_ expressed as kg N ha^-1^ yr^-1^) with a saturating curve limited by *N*_*addmax*_ representing the maximum asymptotic response to nitrogen addition [40]. A saturating exponential function was chosen to describe the N addition effect:

$$N_{add}=N_{add\max}\times (1-a^{N_{amount}/30})$$ (3)

where *a* is a constant (set to 0.75 here) and *N*_*add max*_ is set to 0.6. With those parameter values, leaf photosynthesis is increased by about 60% under very high nitrogen supply, which is in agreement with the positive effects of nitrogen addition on grassland productivity [36, 37].

### Biological potential productivity and grass-fed livestock density

ORCHIDEE-GM simulates two options, through which managed grassland provides forage for livestock: grazing and mowing. Under grazing conditions, thresholds of shoot biomass are set for starting, stopping and resuming grazing [28], while under mowing, the frequency and magnitude of forage harvests in each grid cell is a function of grown biomass [28], and the model carbon dynamics equations then calculate the potential productivity. Here, we define grassland productivity as the annual production of forage from cut grassland. Simulated productivity can be validated against the variable names *yields* in the European Eurostat statistics at the scale of administrative units (Eurostat Agriculture, forestry and fisheries; referred as Eurostat-AFF [41] hereafter). Spatial statistical information on stocking rates (grazing-animal density) and on the ratio between cut and grazed areas in each grid cell is not available at European scale. Thus a set of rules describing an idealized self-sufficient herbage-based ruminant livestock farm [28] has been introduced into ORCHIDEE-GM. These rules are based upon three assumptions, applied for each grid cell, 1) livestock is only fed by herbage (i.e. arable crop-feed products are not considered), 2) grazing occurs throughout the vegetation period, and that mown biomass must satisfy the feed requirements of the animals outside the growing season, and that no lateral herbage (e.g. hay) goes in or out of the grid cell, and 3) the use of grassland production is maximized by determining a combination of grazing and mowing, that maximizes the number of animals. Our model thus calculates how much livestock may potentially be fed by grass biomass in each grid cell. Accordingly, we call the results of the model built upon these rules, *potential* or *optimal* productivity and animal stocking rate.

Under these assumptions, the optimal animal stocking rate, *S*_*opt*_ (number of livestock units (LSU) per hectare) and the optimal proportion of grazed versus cut grasslands, *F*_*opt*_ (within [0,1]) are calculated for each grid cell. The annual herbage production, *Y*_*cut*_, (kg of dry matter (DM) per hectare) of cut grasslands that occupy a fraction (*1-F*_*opt*_) of a grid cell should be equal to the herbage dry matter required by herbivores under cover (Eq 4), while the production under grazing (*Y*_*graze*_) needs to meet dry matter requirements during the vegetation period (Eq 5). Due to the impact of livestock on grass growth through trampling, defoliation etc., and because grassland cannot be continuously grazed during the vegetation period, the effective yield on grazed grassland depends on the stocking rate and on the environmental conditions of the grid cell (Eq 6). Given these interdependencies, *S*_*opt*_ and *F*_*opt*_ are calculated using the optimization algorithm of Vuichard et al. [28]. The ‘recovery’ time required under grazing is obtained in the model using threshold, which determine when grazing stops (dry biomass remaining lower than 300 kg DM ha^-1^), or when grazing can start again (dry biomass recovered to a value above 300 kg DM ha^-1^ for at least 15 days).

$$IC\times T_{farm}\times S_{opt}\times F_{opt}=Y_{cut}\times (1-F_{opt})$$ (4)

$$IC\times (1-T_{farm})\times S_{opt}\times F_{opt}=Y_{graze}\times F_{opt}$$ (5)

$$Y_{graze}=f\{\begin{array}{cc} grid & cell \end{array},S_{opt}\}$$ (6)

where IC is the daily intake capacity (with a mean value of 13 kg DM LSU^-1^ day^-1^, assuming 1 LSU is the grazing equivalent of one adult dairy cow producing 3000 kg of milk annually, without additional concentrated foodstuffs [42]; using typical digestible energy expressed as a percentage of gross energy (DE%: 45%- 55%) suggested by IPCC guidelines [43], IC ranges from 12–14 kg DM LSU^-1^ day^-1^ calculated based on Eq 10.18b in IPCC guidelines [43]) of animals during grazing and under cover, *T*_*farm*_ is the number of non-growing season days during which herbivores need to be fed with cut herbage. The potential animal density (*D*_*opt*_) is given by:

$$D_{opt}=S_{opt}\times F_{opt}$$ (7)

*D*_*opt*_ is calculated in the iterative algorithm by increasing the input animal stocking rate (*S*_*opt*_) until convergence is reached and the stocking rate reaches its potential value and cannot increase further. *D*_*opt*_ must be interpreted as a potential herbage-only limited (i.e., grass-fed) livestock density of a given model grid cell (similar to the term “livestock carrying capacity” [44]). When *D*_*opt*_ is reached, the grassland herbage production is fully used by livestock and the herbage intake capacity of the livestock is reached. The model assumes that the grassland herbage is highly digestible, i.e., dominated by leafy herbage with a low proportion of stem and dead material, which is usually not the case in rough pastures [7]. Furthermore, other limiting factors, such as low soil pH, low soil nitrogen mineralization and low soil phosphorus, the density of unpalatable plants that animals refuse to eat, soil degradation and slope are not taken into account. Therefore, the actual grass-fed livestock density tends to be lower than *D*_*opt*_ because those factors reduce grassland productivity. On the other hand, the use of additional arable crop-feed diet supplements justified by economic considerations (e.g., estimated in [3]), for instance in the production of high economic value dairy products, may locally increase the actual livestock density above *D*_*opt*_.

### Modeling adaptive management in response to climate variability and CO_2_

**Fig 1:**
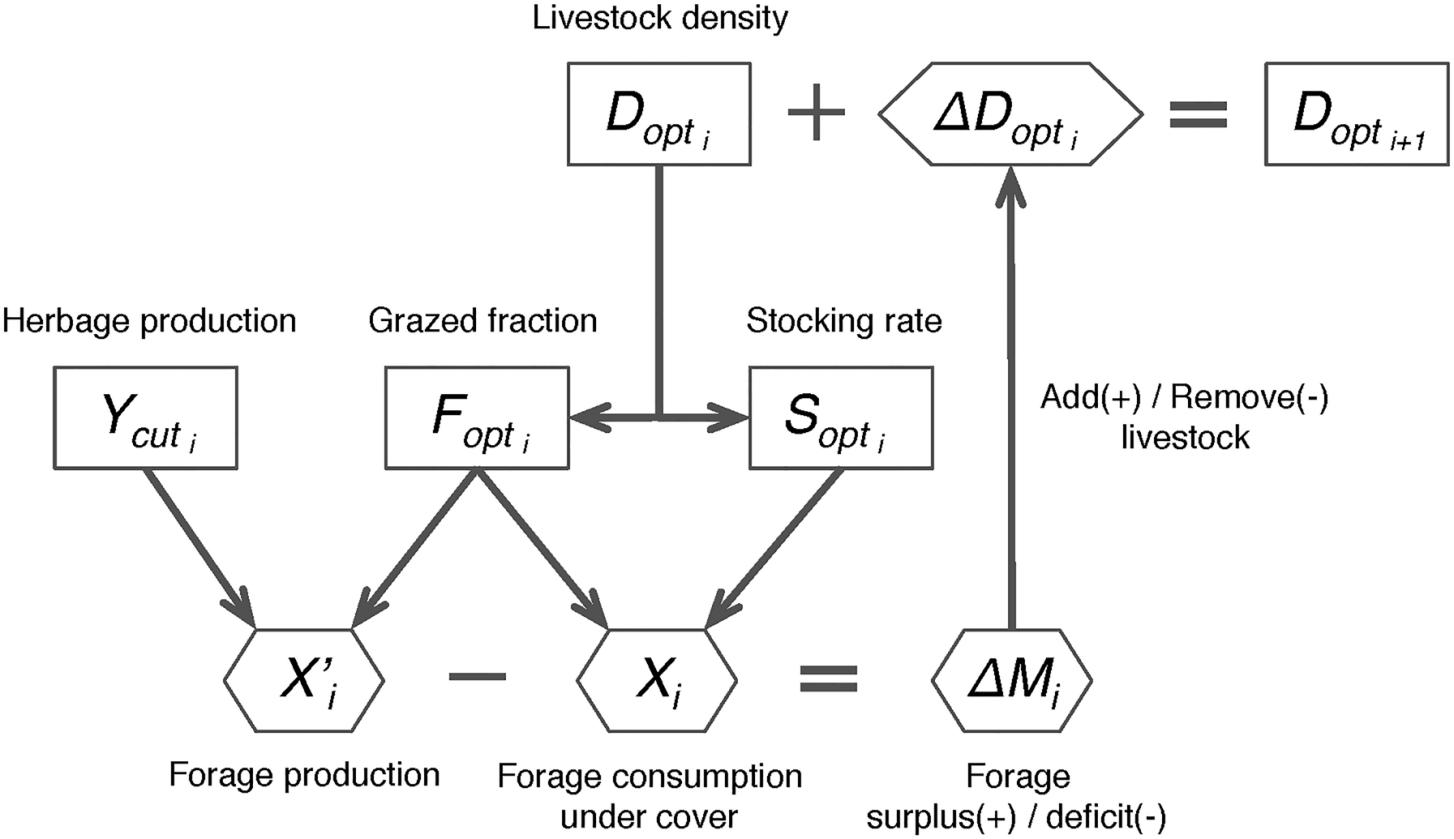
Strategy for modeling adaptive management in ORCHIDEE-GM. See text for symbol definitions.

The algorithm developed by Vuichard et al. [32] is designed to define the optimal number of livestock (*D*_*opt*_) in equilibrium with a given climate condition. We then improved the algorithm to be able to simulate the progressive adaptation of livestock density to climate change. New rules were defined and incorporated into ORCHIDEE-GM to account for how grassland management might change in response to a climate-driven change in productivity. We start from animal stocking rate (*S*_*opt*_) and proportion of grazed grasslands (*F*_*opt*_) optimized for a productivity in equilibrium with a given climate condition. In the case productivity changes (e.g., because of climate change), the profit-driven farmers will tend to fully use the herbage production from the farm, and in practically, take adaptive actions to restore the biological potential grass-fed livestock density by changing livestock numbers (Fig 1). Specifically, at the end of a given year, *i*, we model that the farm manager in each grid cell takes the decision to add (in case of forage surplus) or to remove (in case of forage deficit) animals to or from the grid cell for the next year, thus changing the previous optimal animal density (*D*_*opt*,*i*_) by a step Δ*D*_*opt*,*i*_ as function of the cut forage surplus or deficit (Δ*M*_*i*_). The new optimal animal density for the next year *i+1* (*D*_*opt*,*i+1*_) is given by:

$$D_{opt_{i+1}}=D_{opt_{i}}+\Delta D_{opt_{i}}$$ (8)

where *D*_*opt*,*i+1*_ is adapted in the model by changing *F*_*opt*_ (in Eq 7), and the step density change (Δ*D*_*opt*,*i*_) is given by:

$$\Delta D_{opt_{i}}=\Delta D_{opt}\times \alpha =\Delta M_{i}/IC/T_{year}\times \alpha$$ (9)

where *IC* is the daily intake capacity, *T*_*year*_ = 365 days. Δ*D*_*opt*,*i*_ is the maximum animal density change resulting from the forage surplus (or deficit) of the previous year (Δ*M*_*i*_), and α is the fraction of Δ*D*_*opt*_ that is realized by the farmer. Given the highly variable productivity caused by the year-to-year variability of climate, the farmers may face a very high risk by adjusting each year their livestock numbers to productivity variations (the situation of α is 100%). For example, in a situation of a extreme drought year *i+1* follows a very productive year *i*, farmers will suffer a heavy loss in year i+1 due to their acquisition of too many animal based on the productivity of year *i*. Therefore, the farmers were assumed to be able to search a balanced response to productivity change between the profit (e.g., get more profit from productivity increase) and the risk (year-to-year variability of productivity). We assumed they tend to choose a reasonable “moderate risk”, which is the response to the productivity change during a relatively long period (e.g., previous 5-years’ mean productivity, corresponding to the α of 20% set in this study). Δ*M*_*i*_ is given by:

$$\Delta M_{i}=X\prime_{i}-X_{i}$$ (10)

$$X\prime_{i}=Y_{cut_{i}}\times (1-F_{opt_{i}})$$ (11)

$$X_{i}=IC\times T_{farm_{i}}\times S_{opt_{i}}\times F_{opt_{i}}$$ (12)

where, in each grid cell, *X’*_*i*_ is the annual total forage grass production from cut grasslands for the year *i*, calculated from Eq 11, and *X*_*i*_ is the herbage dry matter required by herbivores under cover during the non-growing season for the year *i*, calculated from Eq 12.

### Simulation set-up

ORCHIDEE-GM is integrated on a grid over Europe using the harmonized climate forcing data from the ERA-WATCH reanalysis for the period 1901–2010 and at a spatial resolution of 25' by 25' [45]. Mean and standard deviation of the ERA-Interim time series [46] were adjusted according to the WATCH time series [47, 48] by using the overlapping period 1989–2001. The harmonized dataset was spatially downscaled to 25' by overlapping CRU CL2.0 [49] monthly means to the spatial anomaly of the harmonized datasets for each single climatic variable. An altitude-based correction was applied for downscaling surface pressure according to a digital elevation map from CRU CL2.0. This resolution (25' by 25') is sufficient to represent regional meteorological regimes accurately in low lying regions, but not in mountainous areas.

Gridded mineral fertilizer and manure nitrogen application rate for European grasslands in the European Union (EU27) was estimated by CAPRI model (see [50, 51]) based on combined information from official and harmonized data sources such as Eurostat, FAOstat and OECD, and spatially dis-aggregated using the methodology described in Leip et al. [52]. The data are estimated at a spatial resolution of clusters of 1 km by 1 km and were re-aggregated here to a spatial resolution of 25' by 25' (S1 Fig). For French regions, we use data from the French national statistics [53]. A set of rules was used to rebuild the temporal evolution of gridded nitrogen fertilization from 1901 to 2010: 1) organic fertilizer is assumed to have remained constant over time; 2), the application rate of mineral fertilizer evolved with time following the total mineral nitrogen fertilizer consumption of European Union [54]; 3) mineral fertilizers are set to be applied since 1951, and application rates linearly increased from 0 to the observed level of 1961 during the period 1951–1960. Besides nitrogen fertilizer application, nitrogen deposition was considered as nitrogen addition as well. Gridded nitrogen deposition rates for Europe was originated from European Monitoring & Evaluation Programme (EMEP) dataset, and was a product of EU-PF7 project (GHG-Europe; data are available at http://gaia.agraria.unitus.it/ghg-europe/data/others-data) where the decadal means were linear interpolated to annual values. It is noteworthy that the N fixation by legumes was not accounted for in this study.

**Fig 2:**
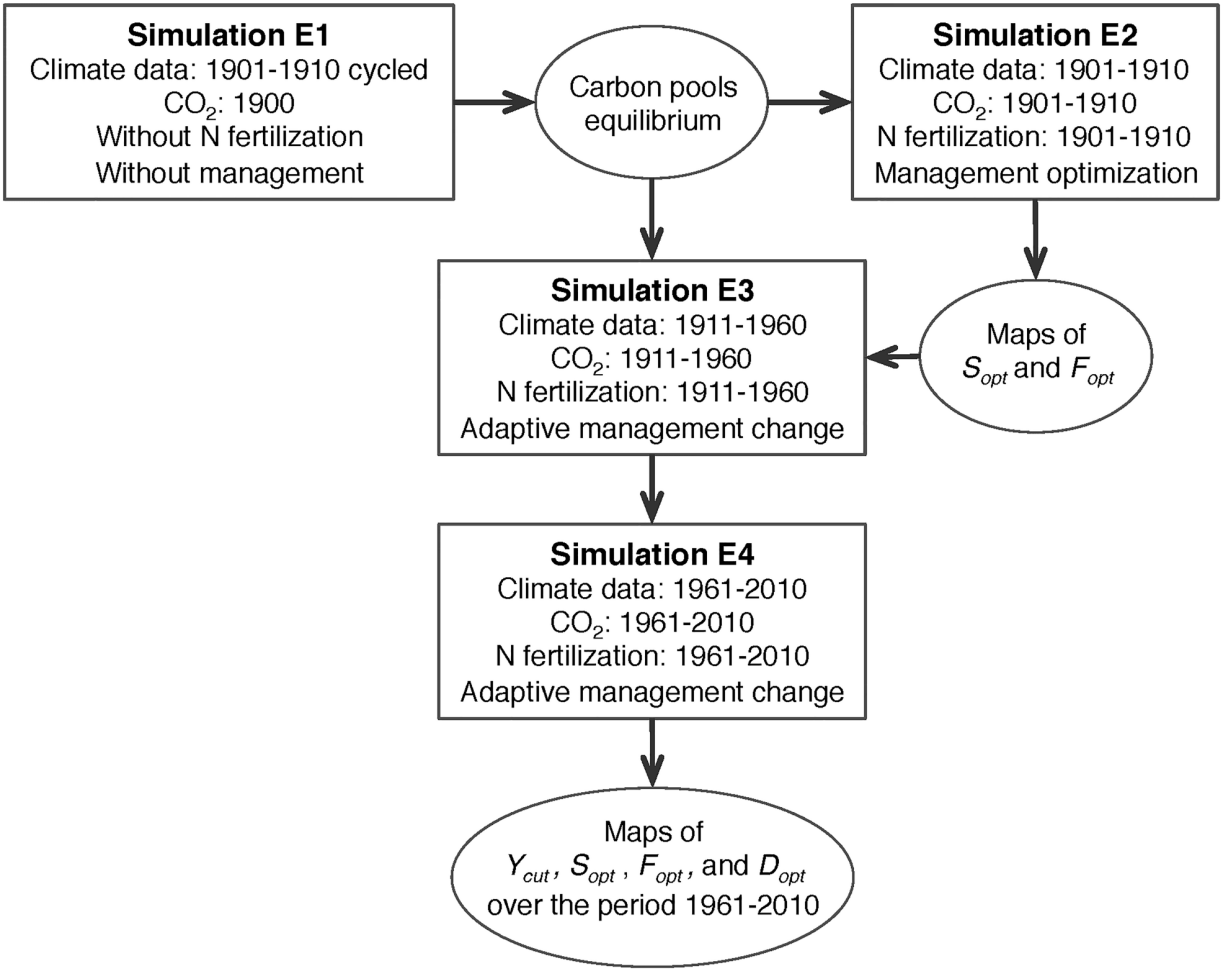
Illustration of the simulation protocol, forcing data and initial state for various simulations.

A series of simulations was carried out, and the illustration of the simulation protocol is shown in Fig 2. ORCHIDEE-GM was first run for a spin-up (simulation E1) without management using the first 10 years of the period 1901–1910 recycled in a loop, and atmospheric CO_2_ concentration for 1900 (296 ppm) until all carbon pools reached equilibrium (long term Net Ecosystem Exchange, NEE = 0 at each grid point). This first spin-up usually takes 10,000 years. Starting from the end of this first spin-up, two separate transient simulations were performed. In the first one (simulation E2), optimal animal stocking rates (*S*_*opt*_) and fractions of grazed grassland (*F*_*opt*_) for the period 1901–1910 were defined by running ORCHIDEE-GM with its optimization algorithm that maximizes stocking rates in each grid cell (see above). In the simulation E3, ORCHIDEE-GM was run during the historical period (1911–1960) with increasing atmospheric CO_2_, variable climate, and with the new climate-adaptive management change algorithm. This transient E3 simulation started with the reference distributions of *S*_*opt*_ and *F*_*opt*_ over Europe (obtained in simulation E2), as well as with soil carbon pools for the year 1910 (end of the spin-up simulation). Starting from the end of simulation E3, a transient simulation E4 was carried out for the period 1961–2010 with increasing atmospheric CO_2_, variable climate, and with the adaptive management change algorithm.

Three further simulations (E5, E6 and E7) investigated the relative contribution of atmospheric CO_2_, climate change and nitrogen fertilization trends on the estimated trend in productivity. The simulations E5, E6 and E7 are the same as simulation E4, but with atmospheric CO_2_ concentration fixed at the level of 1961 (E5), using the first five years of climatology data (1961–1965) recycled (E6), or with nitrogen fertilization and nitrogen deposition fixed to the level of 1961 (E7), respectively. The difference in productivity trend between simulations E4 and E5 reflects the effects of increased CO_2_. The effects of climate variation and nitrogen fertilization were derived as the difference between simulations E4 and E6 and between simulations E4 and E7, respectively.

### Regional census statistics (grassland area, livestock numbers)

The European statistical database, Eurostat [41], divides the European Union (EU) into regions at three different levels abbreviated as NUTS (Nomenclature des Unités Territoriales Statistiques [55]). Above NUTS-1 (referred as *NUTS-0* hereafter) is the country level of the Member State. Eurostat [41] provides data on grassland area and livestock numbers for NUTS regions [55]. A full scope Farm Structure Survey (FSS; Council Regulation (EEC) No 571/88, and Regulation 1166/2008) is carried out every 10 years since 1990, with sample surveys every 2 or 3 years. In this study, we compiled data from 30 countries (EU28 plus Norway, and Switzerland) and 279 regions (NUTS-2 level except for Germany, where only NUTS1-level data are available) of Europe. Eurostat statistics divide grassland into classes: *temporary grassland* (productive grassland less than five years after sowing), *permanent grassland* (productive grassland, five years or more after sowing), and *rough grazing* (low productivity pasture, extensively grazed, often in fragile areas). Ruminant livestock of various species and ages are aggregated into livestock units (LSU), by applying specific coefficients initially established on the basis of the nutritional or feed requirement for each type of animal. Livestock unit coefficients are derived from Eurostat [42] (e.g., 0.1, 0.8 and 1 LSU per head for sheep and goats, and heifers and dairy cows, respectively).

### Data for evaluating the simulated productivity of European grasslands

Smit et al. [20] constructed a map of Europe showing the spatial distribution of grassland productivity by integrating census statistics, literature, and expert judgment using the NUTS classification [55]. The biological potential of grassland productivity from ORCHIDEE-GM (on a spatial resolution of 25') was aggregated to the NUTS-2 level weighted by the corresponding grassland area in each grid cell (from CORINE Land Cover map, *CLC2000* [56]). We then compared the spatial pattern of modeled potential productivity with the map of Smit et al. [20] averaged over the period 1995–2004.

Net primary productivity (NPP), including above- and below-ground plant organs, represents the net flux of carbon from the atmosphere into plants per unit time (one year in this study). NPP is a crucial variable in vegetation models. However, high quality measurements of grassland NPP are sparse due to the difficulty of measuring some NPP components such as fine-root production [57]. A new version of the Luyssaert et al. [58] database comprising non-forest biomes (Campioli, unpublished) was obtained. NPP data from seven European temperate grassland sites of this database were compared with NPP of cut grassland simulated with ORCHIDEE-GM at their corresponding locations on the grid.

The temporal evolution of grassland productivity is not available for most regions. However for some countries, the Eurostat statistical database provides long-term grassland yield data from 1973 onwards, which are directly calculated as harvested production per unit area [59]. NDVI has been shown to be positively correlated with temperate grassland productivity [60]. We therefore used the NOAA/AVHRR NDVI composites at a spatial resolution of 8-km and 15-day intervals produced by the Global Inventory Modeling and Mapping Studies (GIMMS) [61] from 1982 onwards. Summer NDVI (June, July and August, JJA) of grid cells dominated by grassland (cells with more than 50% of the area are attributed to grassland in CLC2000 land cover map) was extracted from GIMMS and then aggregated to NUTS-0 level for the countries with long-term grassland productivity data from Eurostat. The time series of grassland productivity statistics (aggregated over the NUTS regions) were compared with the modeled productivity of cut grassland (see above), and grassland NDVI was correlated with aggregated modeled productivity in the 25 km grid cells corresponding to those used for NDVI.

### Livestock distribution and its ratio to productivity

FAO [62] provides a 5' by 5' global livestock distribution maps for major animal species (cows, pigs, poultry, sheep, goats, and buffalo), which are consistent with regional statistics. Cattle, sheep and goat density is expressed in this study as the number of animals (head) per square kilometer of land suitable for livestock production [62]. A ruminant livestock density in LSU ha^-1^, was calculated from these data by using head-to-LSU conversion factors of 0.8, 0.1, and 0.1 for cattle, sheep and goats, respectively. This LSU density distribution was then aggregated to the 25' by 25' grid used by ORCHIDEE-GM. We also aggregated the livestock density distribution from FAO to the NUTS-2 level, for comparison with grassland productivity statistics from Eurostat summarized by Smit et al. [20]. Then, the average ratio of FAO livestock density to grassland productivity from the Eurostat data was calculated in each NUTS region.

### Grass-fed livestock numbers estimated from statistics and model simulations

In Europe, ruminant livestock are not only fed on grass, but also receive crop-feed and crop by-products. Thus, the number (LSU) of grass-fed livestock (*N*_*obs*_) in each region can be calculated separately as:

$$N_{obs}=N_{beef}\times F_{beef}+N_{dairy}\times F_{dairy}+N_{sheep}\times F_{sheep}+N_{goats}\times F_{goats}$$ (13)

where *N*_*beef*_, *N*_*dairy*_, *N*_*sheep*_ and *N*_*goats*_ are the actual numbers (LSU) of beef cattle, dairy cattle, sheep, and goats given by regional census statistics; *F*_*beef*_, *F*_*dairy*_, *F*_*sheep*_ and *F*_*goats*_ are the fractions of grass-feed in the diet of each type of animal, available from the study by Wirsenius [3] (differentiating Western and Eastern Europe; S1 Table).

ORCHIDEE-GM simulates the potential livestock density *D*_*opt*_ on a 25 km × 25 km grid over Europe. This model output was re-aggregated to the NUTS level (*D*_*reg*_, for NUTS-0 to NUTS-2) and weighted by the actual grassland area of each grid cell from the CORINE Land Cover map (*CLC2000* [56]). The simulated number of grass-fed livestock (*N*_*sim*_) is given by:

$$N_{sim}=f_{temp}\times D_{reg}\times A_{temp}+f_{perm}\times D_{reg}\times A_{perm}+f_{rough}\times D_{reg}\times A_{rough}$$ (14)

where A_temp_, A_perm_, and A_rough_ are the area of *temporary grassland*, *permanent grassland*, and *rough grazing* respectively in each NUTS region (data from Eurostat; names in italics from Eurostat terminology, see above); *f*_*temp*_, *f*_*perm*_, and *f*_*rough*_ are the fraction of *D*_*reg*_ that grassland of different types are assumed to support, and are set to 100%, 80%, and 10% for *temporary grasslands*, *permanent grasslands*, and *rough grazing* lands respectively according to their productivity. Newly sown *temporary grassland* has high productivity, and thus is assumed to support the simulated potential livestock density, whereas less productive *rough grazing* grasslands are assumed to receive little management and to support only 10% of *D*_*reg*_ (these grasslands are usually located in mountainous areas with steep slopes and limited accessibility). Combining modeled livestock density with statistical grassland-area data, we produced a simulated number of animals for each region, *N*_*sim*_ that can be compared with *N*_*obs*_ available from the statistics.

Long-term regional time series of grassland area and livestock numbers (*N*_*obs*_) are not available for the Eurostat NUTS regions. Therefore, country average *N*_*obs*_ (also called NUTS-0) from 30 countries from the FAO statistical database [5] were used to evaluate modeled trends of *N*_*sim*_ over the period from 1961 to 2009.

All the data used in this study are listed in Table 1, as well as the corresponding model results for comparison.

**Table 1:** Model results and data used for comparison.

Model results	Data	Source
Grassland productivity	Statistical productivity	[41], assembled by Smit et al. [20]
	GIMMS NDVI	[61]
Net primary productivity (NPP)	Site NPP observations	NPP database (Campioli, unpublished)
Livestock density	Global livestock distribution maps	FAO [62]
Regional grass-fed livestock numbers	Grassland area	[41]
	Livestock numbers	[41]
	Livestock diet	[3]

### Model-data agreement metrics

To assess model-data agreement for the spatial distribution of grassland productivity, we use the Pearson’s product-moment correlation coefficient (*r*) with P-value. *r* quantifies the proportion of the total variance in the observed data that can be explained by the model, given by:

$$r=\frac{\sum_{i=1}^{n}\left(P_{i}-\bar{P})(O_{i}-\bar{O}\right)}{\sqrt{\sum_{i=1}^{n}{\left(P_{i}-\bar{P}\right)}^{2}}\sqrt{\sum_{i=1}^{n}{\left(O_{i}-\bar{O}\right)}^{2}}}$$ (15)

where *P*_*i*_ is modeled data, *O*_*i*_ is observed data, $\bar{\text{P}}$ is modeled mean, $\bar{\text{O}}$ is observed mean, and n is the number of data-points. The P-value is the probability that the correlation coefficient is zero (null hypothesis). When the P-value is lower than the conventional 5% (P < 0.05) the correlation coefficient is statistically significant. The correlation coefficient (*r*) is also used with the P-value to assess the model-data agreement on the interannual variability of productivity time series (including observed and modeled productivity and summer NDVI). For all time series, model output, statistics and summer NDVI), long-term trends were removed (detrended).

To evaluate grass-fed livestock numbers in Europe, ordinary least squares linear regression with intercept forced to zero is performed between *N*_*sim*_ and *N*_*obs*_. A regression coefficient (*slope*) close to unity with a high coefficient of determination (R^2^) and with P < 0.05 indicates a good model-data agreement.

All the statistics in this study were calculated using Interactive Data Language (IDL) software (Version 8.3 (linux x86_64 m64). (c) 2013, Exelis Visual Information Solutions, Inc. http://www.exelisvis.com/ProductsServices/IDL.aspx).

### Uncertainties of the potential grassland productivity estimation

The uncertainties of predictions from process models could be rather large resulted from model forcing data [63, 64], parameter values uncertainty [65], as well as model structure related uncertainty [66–68]. At a large geographical scale (Europe), a comprehensive assessment of uncertainty, such as using factorial designs [69] and Monte Carlo-type stratified sampling approach [70], requires many model runs and are therefore prohibitive for complex models (such as ORCHIDEE-GM) with a large number of parameters, a half-hourly time step and a high computational demand [71]. In this study, we identified four model parameters that likely substantially contribute to uncertainties of productivity simulations [63, 65, 69, 72–74]. These four sources of uncertainties, define 16 combinations given minimum and maximum values that define a range (± 20% approximately) around the standard values used in the control simulation. The uncertain settings that are tested by sensitivity simulations are (1) the constant *a* (Eq 3) determining the shape of saturating curve, (2) the response of photosynthetic capacity to nitrogen addition (parameter *Nadd*_*max*_, Eq 3), (3) the maximum rate of Rubisco carboxylase activity (*Vcmax*_*opt*_) and the maximum rate of photosynthetic electron transport (*Jmax*_*opt*_), and (4) the prescribed maximum specific leaf area (*SLA*_*max*_ [29]). Simulations with the 16 factors combinations at the full geographical scale of this study (9237 grids) would require enormous computational time beyond our capability. Thus, evenly spaced 368 grid cells over our study area were selected for the uncertainty analysis, which were able to well represent the spatial distribution, magnitude and interannual variability of grasslands’ productivity (see S1 File for detail). Complete simulations (as described in section “Simulation set-up”) were conducted at these grids with these factors combinations (with minimum and / or maximum values for each factors; Table 2). The standard deviation (SD) of the simulated results is used to characterize and assess the uncertainties of potential productivity, grass-fed livestock density, and their trends.

**Table 2:** Key model parameters for grassland productivity simulations and their ranges.

Model Parameters	Unit	Standard value	Minimum	Maximum	Description
*a*	unitless	0.75	0.6	0.9	proportions of intensively managed grasslands
*N*_*addmax*_	percent	60%	40%	80%	the saturate status of N addition effect on photosynthetic capacity
*Vcmax*_*opt*_ */ Jmax*_*opt*_	μmol m^-2^ s^-1^	55 / 110	44 / 88	66 / 132	*Vcmax*_*opt*_: the maximum rate of Rubisco carboxylase activity *Jmax*_*opt*_: the maximum rate of photosynthetic electron transport
*SLA*_*max*_	m^2^ gC^-1^	0.048	0.0384	0.0576	the prescribed maximum SLA

Note: Factors are modified by ±20% of standard value (except for *N*_*addmax*_, which was modified by ± 20% of absolute value). For each combination, minimum or maximum value of each factor is used, which forms 2^4^ = 16 factor combinations.

## Results

### Distribution of potential grassland productivity

**Fig 3:**
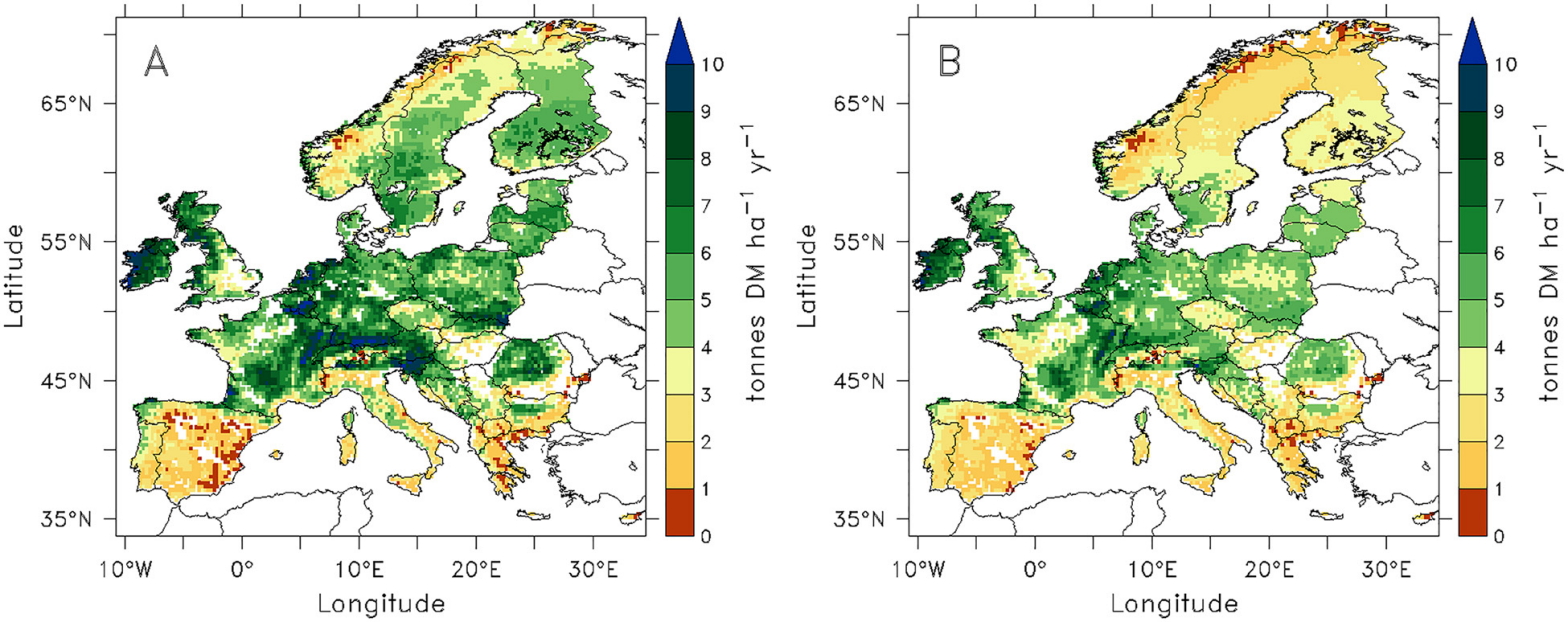
Biological potential productivities simulated by ORCHIDEE-GM of (A) potential net primary productivity (harvested dry matter in forage) in cut grasslands and of (B) potential net primary grazed dry matter used for animal intake in pasture. Both fields are average values for the period 1995–2004.

ORCHIDEE-GM (Fig 3A) simulates the highest potential grass production (annually harvested forage biomass per unit of cut grassland area) in the Atlantic maritime-climate zone (The Netherlands, Belgium, Luxembourg, and west of Ireland) with values reaching up to 10 tonnes DM ha^-1^ yr^-1^, and in the lower elevation parts of the Alps (e.g., southern Germany, west of France, northern part of Austria and Switzerland, northeast Italy, and Slovenia). The model simulates a lower productivity in Mediterranean countries (e.g., Greece, southern Italy, Spain, and Portugal) and in Scandinavia (e.g., Norway, northern Sweden and northern part of Finland,) and in the high altitude grid cells of the Alps. The modeled distribution of biomass eaten by grazing animals follows the same pattern as the production of harvested forage grass (see Fig 3B). However, biomass grazing amounts to only about 78% of harvested forage biomass. The difference comes from the lower herbage use efficiency of grazing (28% of NPP) compared with cutting (40% of NPP), given the similar NPP values simulated by ORCHIDEE-GM for cut (508 g C m^-2^ yr^-1^) and grazed grasslands (577 g C m^-2^ yr^-1^).

**Fig 4:**
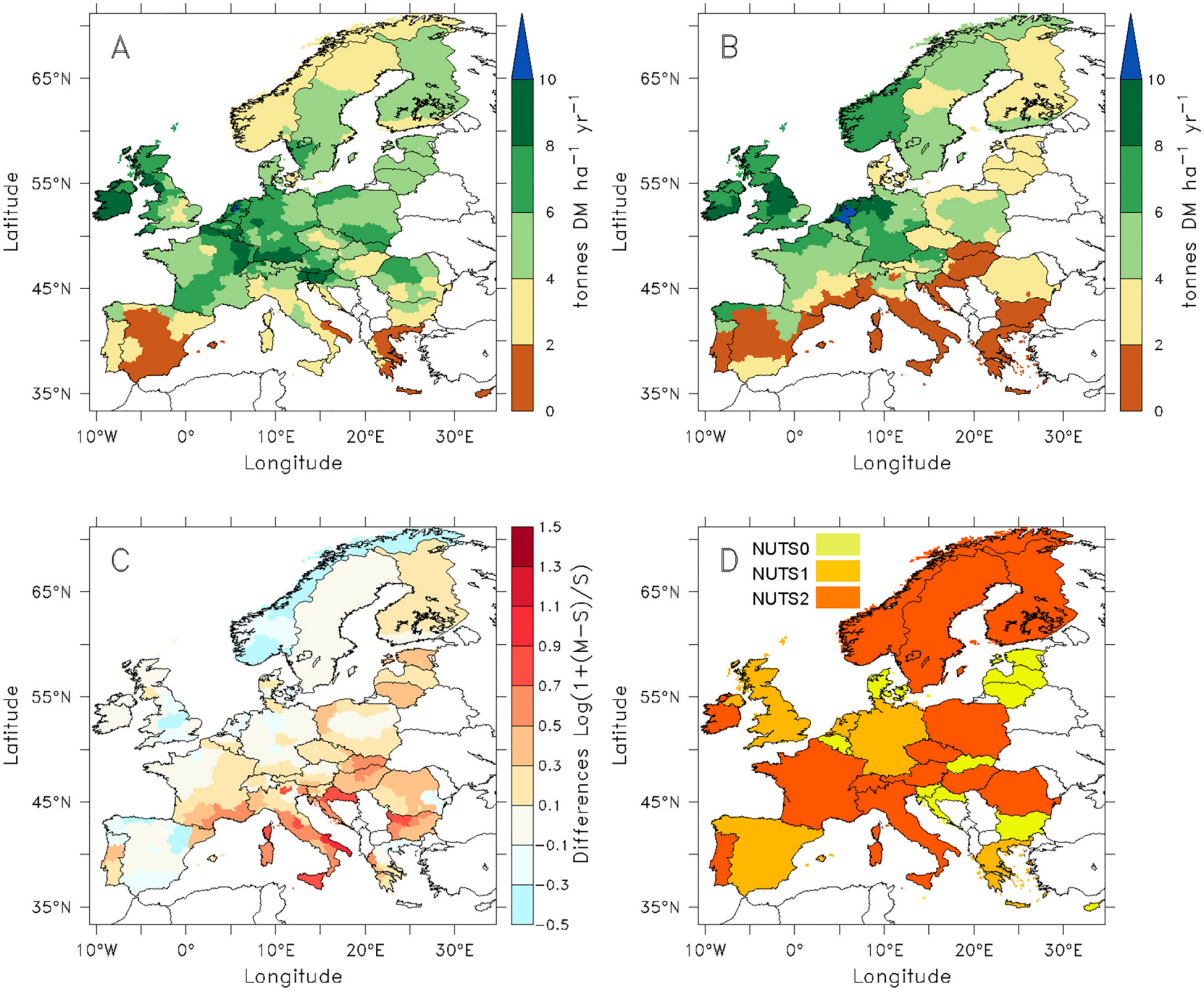
Spatial distribution of (A): potential grassland productivity simulated by ORCHIDEE-GM from cut grasslands (M), (B): actual grassland forage productivity from Smit et al. [20] (S), (C): relative discrepancy between them expressed as log (1+(M-S)/S), and (D): the NUTS administrative units at which statistical data are available. Simulated and “observed” data from statistics represent both a 10-year average from 1995 to 2004.

When the simulated productivity at the pixel level is aggregated over the Eurostat administrative regions (NUTS-2 to country level, depending on data availability, see Fig 4D), a significant positive spatial correlation (*r* = 0.6; P < 0.01) is obtained between simulated and observed productivity across the 167 NUTS regions. However, ORCHIDEE-GM tends to simulate higher potential productivity than the actual productivity (yield) reported in the Smit et al. [20] in most regions (Fig 4). This result is logical because the model simulates the potential (maximum) productivity of permanent cut grassland, whereas Eurostat productivities are based on actual harvest data. Exceptions are northern Spain, Norway, and northern Sweden where ORCHIDEE-GM simulates lower productivity than that from statistics. The positive difference between simulated potential and actual productivities [20] (Fig 4C) is the largest for Mediterranean (e.g. southern France and southern Italy) and east European regions.

Observed and modeled NPP are comparable (R^2^ = 0.76, P < 0.001, Table 3), showing that ORCHIDEE-GM is well able to reproduce the NPP of productive grasslands without nitrogen limitation (e.g. sites CZ-mok and IT-bea; see list in Table 3), whereas it tends to overestimate NPP (up to 1.6-fold) at sites with relatively low productivity. However, given the fact that some local conditions (such as soil properties, topographic features, etc.) are not considered in the model and that few observations are available for this exercise, any conclusions on the bias of the model in simulating NPP can only be drawn with low confidence.

**Table 3:** Comparison of observed and modeled annual NPP at seven temperate grassland sites.

Site ID	Latitude	Longitude	Altitude (m)	Year	Observed NPP (g C m^-2^yr^-1^)	Modeled NPP (g C m^-2^yr^-1^)	References	Vegetation Source
CH-jur	47° 33’ N	07° 34’ E	520	1994	453	694	[95]	Semi-natural calcareous grassland
				1995	554	679	[95]	Semi-natural calcareous grassland
				1996	495	691	[95]	Semi-natural calcareous grassland
CZ-kam	49° 43’ N	15°58’ E	1424	1976	457	539	[96]	Moist meadow
				1977	628	624	[96]	Moist meadow
				1978	428	552	[96]	Moist meadow
				1979	409	596	[96]	Moist meadow
CZ-mok	49° 01’ N	14° 46’ E	430	2007	777	670	[97]	Nutrient-poor wet grassland
				2007	924	938	[97]	Nutrent-rich wet grassland^a^
DE-gri*	50° 57’ N	13° 30’ E	375	2004	403	646	[98]	Temperate grassland
IT-bea*	46° 00’ N	13° 01’ E	43	2007	1159	1025	[99]	N_2_-fixing grassland used as fodder
				2008	1109	976	[99]	N_2_-fixing grassland used as fodder
SE-kje*	60° 10’ N	17° 38’ E	30	1982–1983	765	586	[100]	Meadow fescue ley, lucerne ley^b^
UK-pge	51° 49’ N	0° 21’ W	128	1970–1985	400	513	[101]	Mixed sward

^a^ For the nutrient-rich wet grassland at site CZ-mok and N_2_-fixing grassland at site IT-bea and SE-kje, site simulations were carried out with an addition of 300 kg N ha yr^-1^ assuming there is no N limitation at the sites.

^b^ For the grassland at site SE-kje, 2 NPP observations with meadow fescue ley (740 g C m^-2^yr^-1^) and lucerne ley (790 g C m^-2^yr^-1^) respectively were averaged to compare with result from ORCHIDEE-GM.

### Distribution of livestock density

**Fig 5:**
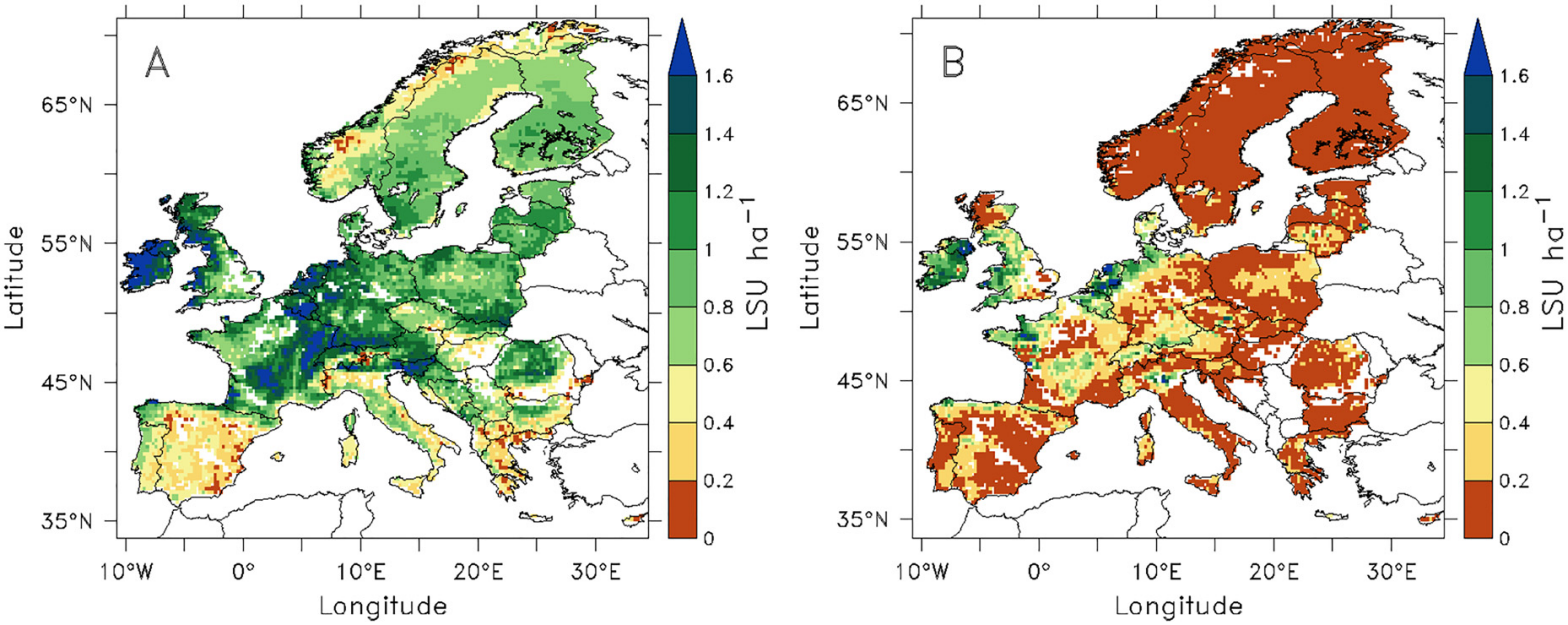
Comparison between (A): potential livestock density from ORCHIDEE-GM expressed in livestock unit (LSU) per ha of grassland, under the modeled optimal cut-grazing management scheme, and (B): observed livestock density from FAO [62].

The modeled potential grass-fed livestock density is compared with the FAO gridded data product [62] in Fig 5. The highest observed densities (> 0.6 LSU ha^-1^) are found in the regions with a maritime climate bordering the North Atlantic and low elevation areas in the Alps; Fig 5B). These two regions are also simulated to have the highest potential livestock density. It should be kept in mind that we model potential livestock densities based on grassland carrying capacity in Europe, whereas the FAO data provide livestock density over land suitable for livestock production (including mosaics of cropland and pasture).

### Distribution of potential mowing frequencies and the length of grazing period

**Fig 6:**
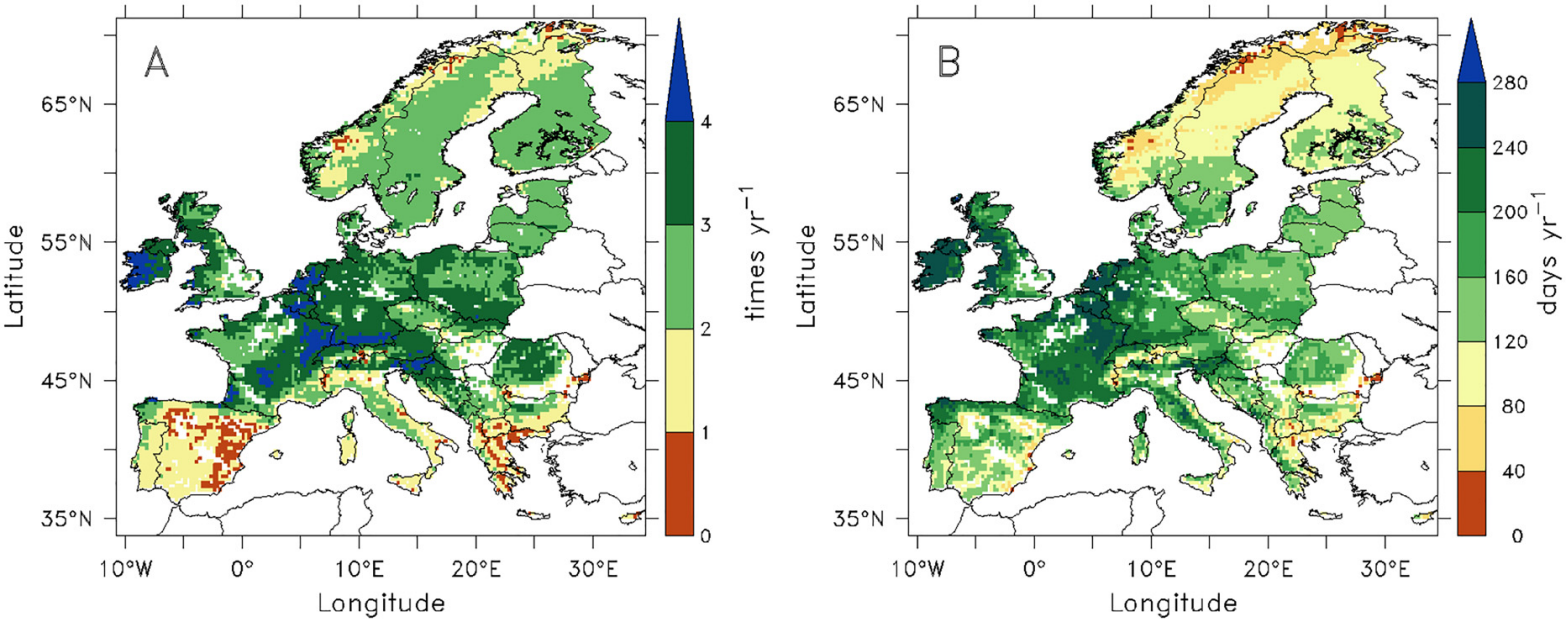
Spatial distribution of potential mowing frequencies (A) and the length of grazing period (B) simulated by ORCHIDEE-GM. Both fields are average values for the period 1995–2004.

Fig 6 shows the spatial distribution of potential mowing frequencies (Fig 6A) and the length of grazing period (Fig 6B) simulated by ORCHIDEE-GM. They follow the same pattern as the production of harvested forage grass and the biomass eaten by grazing animals (Fig 3). In the Atlantic maritime-climate zone (The Netherlands, Belgium, Luxembourg, and west of Ireland) and in the lower elevation parts of the Alps (e.g., southern Germany, west and central part of France, and Slovenia), cut grasslands could be mown as frequent as four times per year, while pasture could be grazed by more than 8 month of a year. Low mowing frequencies (less than 2 times per year) and short grazing season were simulated in Mediterranean countries (e.g., Greece, southern Italy, Spain, and Portugal) and in the mountain area and the high latitude of Scandinavia (e.g., the mountain area of Norway, northern Sweden and northern part of Finland,) and in the high altitude grid cells of the Alps.

### Temporal evolution of grassland production and livestock density

**Fig 7:**
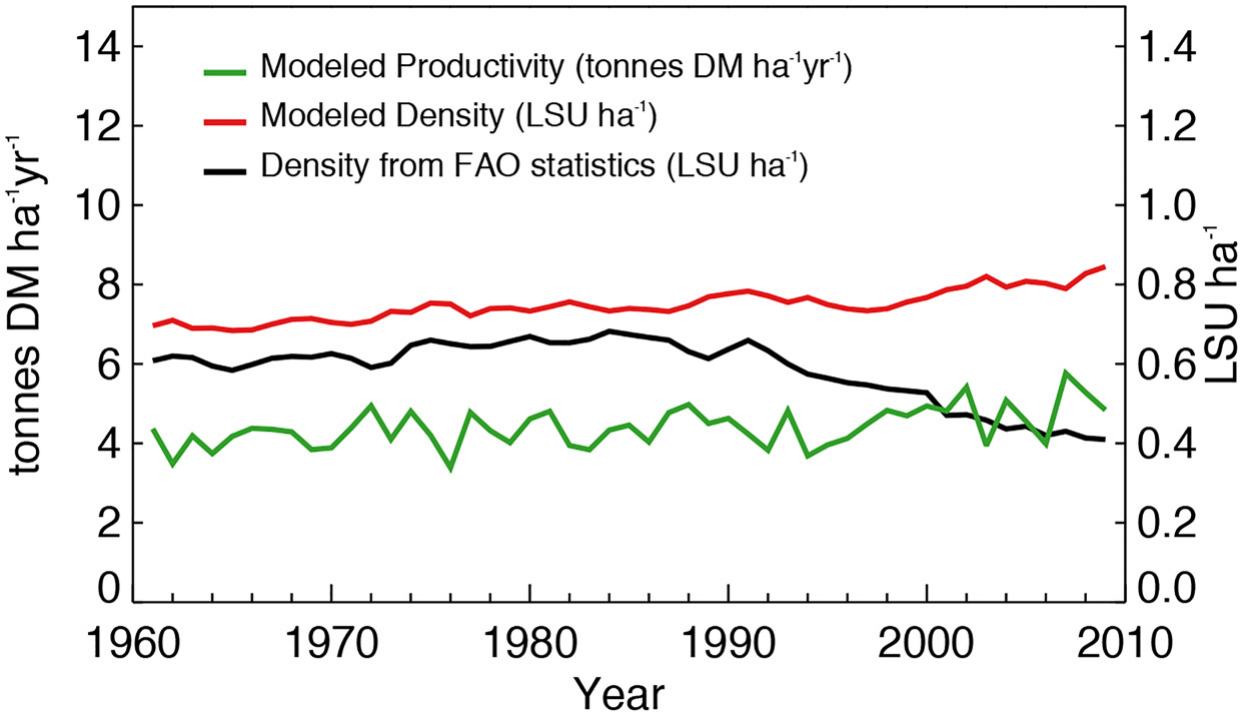
Temporal variations of modeled grassland productivity and of livestock density from model simulations and grassland agricultural statistics (see text) respectively. LSU: livestock unit. All variables are spatial averages over European grasslands masked by CORINE Land Cover map (*CLC2000* [56]).

ORCHIDEE-GM simulates an annual increase (0.36% ± 0.06% per year, P < 0.001) of potential grassland productivity over the last five decades (Fig 7). Because the model mostly neglects other factors, this trend is driven by CO_2_ concentration, climate change, nitrogen fertilizer applications and nitrogen deposition. The relative contribution of these drivers of the trend in potential productivity are 97%, -3% and 15%, respectively (Table 4). The total contribution of the three factors is larger than 100%, because these variables are not independent. The productivity trend is surprisingly close to the mean annual trend in the production of sown grasses estimated from measurements (0.35% per year; reviewed by Smit et al. [20]), although it is unclear whether the observed production gain can be attributed to genetic or to environmental (e.g., atmospheric CO_2_ and climate) factors. A smaller trend was found in the simulated potential livestock density, which increased at a rate of 0.32% ± 0.06% per year (P < 0.001). The livestock density from FAOstat shows a similar but larger trend of 0.33% per year (P < 0.001) between 1961 and 1990, and then a strong decline (2.6% per year, P < 0.001) from 1991. However this decrease in livestock density comes with an increase in milk and meat productivity per head, as a result of the increasing use of crop-feed products including imported feed such as soybean [75]. In 1984 the European Community introduced milk production quotas that contributed to a reduction in the dairy cow population in Europe. This was followed in 1991 by the Nitrates Directive (91/676/EEC) [76] that restricted the application of animal manure in “nitrate vulnerable zones” to a maximum of 170 kg N ha^-1^ yr^-1^, effectively capping livestock density in pastures at some 1.7 LSU ha^-1^. These two policies have caused a decrease in livestock numbers in Europe. The uncertainty of the simulated trends above comes from 1-sigma standard deviation of the trends from 16 sensitivity tests.

**Table 4:** The contribution of rise in atmospheric CO_*2*_, climate change and nitrogen fertilization (including nitrogen deposition) trends on trends in potential productivity.

Productivity trend	Contribution						Total contribution	
T_E4_	Rise in CO_2_		Climate change		N fertilization and deposition trends			
(t DM ha^-1^ yr^-2^)	T_E4_-T_E5_	Percentage	T_E4_-T_E6_	Percentage	T_E4_-T_E7_	Percentage	Value	Percentage
1.62×10^-2^	1.56×10^-2^	97%	-0.05×10^-2^	-3%	0.24×10^-2^	15%	1.76×10^-2^	109%

Note: T_E4_, T_E5_, T_E6_ and T_E7_ are productivity trends from simulation E4, E5, E6 and E7 respectively.

**Fig 8:**
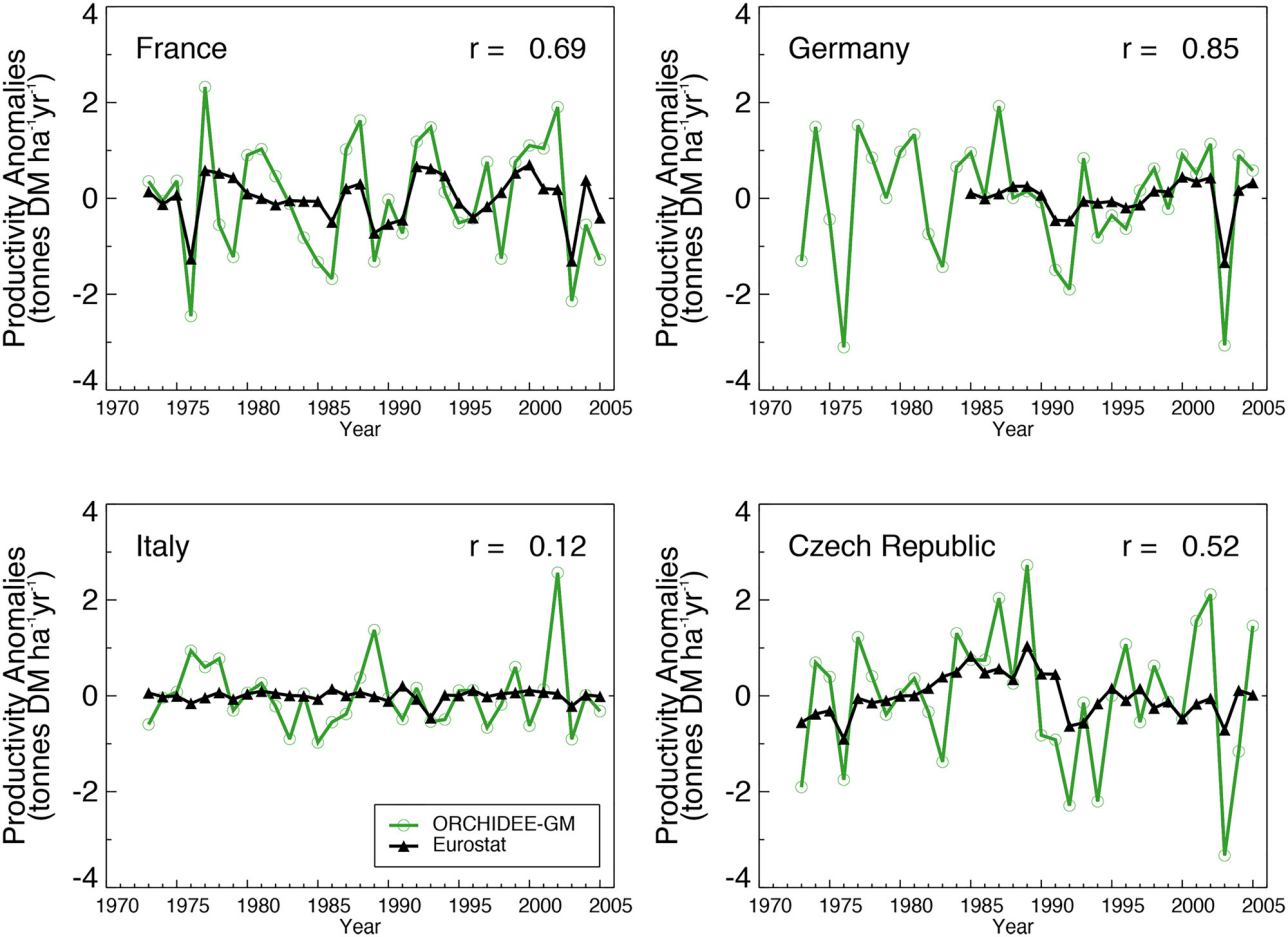
Normalized temporal evolution of modeled and observed productivity of grasslands in four European countries between 1973 and 2005. Observed productivity is derived from Smit et al. [20] based on Eurostat agricultural statistics data (see text).

Changes in grassland productivity between 1973 and 2005 are shown for France, Germany, Italy and the Czech Republic (Fig 8). In Germany, the data show a steep decline of productivity after 1985, which may reflect a change in the definition of pastures [20]. Thus for Germany, only the normalized series after 1985 were shown when statistics are consistent. For the above-listed countries, ORCHIDEE-GM reproduces the drops in productivity observed during the 1976 and 2003 droughts. Further, ORCHIDEE-GM is also able to reproduce the interannual variability of production, although Italy is an exception. High correlations between time series of modeled and observed [20, 41] productivity are found in France (*r* = 0.69, P < 0.01), Czech Republic (r = 0.52, P < 0.01), and the best results in Germany (*r* = 0.85, P < 0.01). The modeled annual productivity is also significantly correlated with summer grassland NDVI in France (*r* = 0.39, P < 0.05), but not in Germany (*r* = 0.31, P = 0.11; Table 5). We use the coefficient of variation (CV) to express the magnitude of the interannual variability in modeled and Eurostat productivities, and the summer NDVI (Table 5). In general, the CV of modeled productivity is larger than the one of Eurostat productivity. The CV of modeled productivity (range 17–30%) is much larger than that of summer NDVI (2–3%).

**Table 5:** Mean value, standard deviation (SD), and coefficient of variation (CV) of modeled and statistical estimates of grassland productivity and NDVI time series for four European countries, as well as correlation coefficient (*r*) between each pair.

	Czech Republic	France	Germany	Italy
Modeled Production (M)				
Mean (t DM ha^-1^ yr^-1^)	5.82	6.41	7.44	2.29
SD (t DM ha^-1^ yr^-1^)	1.50	1.48	1.26	0.70
CV (%)	26	23	17	30
Statistical Production (S)				
Mean (t DM ha^-1^ yr^-1^)	3.26	4.14	6.3	1.54
SD (t DM ha^-1^ yr^-1^)	0.45	0.51	0.47	0.19
CV (%)	14	12	7	12
Summer NDVI (June—August) (N)				
Number of point	3	688	309	18
Mean	0.84	0.82	0.83	0.74
SD	0.02	0.02	0.02	0.02
CV (%)	3	2	2	3
*r*				
M vs. S (1973–2005^a^)	**0.52**	**0.69**	**0.85**	0.12
M vs. N (1982–2010^a^)	0.22	**0.39**	0.31	0.20

Note: To represent interannual variability, long-term trends were removed in all series (detrended) before the calculation of *r*. M vs. S, *r* between modeled and statistical grassland productivity; M vs. N, *r* between modeled productivity and NDVI. *r* values in bold indicate a statistically significant correlation (with P < 0.05). Modeled productivity time series are from 1961 to 2010; statistical productivity time series are from 1973 to 2005 (for Germany from 1985 to 2005); NDVI time series are from 1982 to 2010.

^a^ The periods of time series that use for the calculation of r and P-value.

### Ratio of livestock density to grassland productivity in Europe

Table 6 compares ORCHIDEE-GM estimates and FAO/Eurostat statistics for the ratio of livestock density to grassland production for different Köppen-Geiger climate zones (S2 Fig; derived from Peel et al. [77]). The modeled ratios in different climate zones are very similar (ranging between 1.58 and 1.75 LSU per 10 tonnes of DM, and do not show significant changes over the simulation periods (1961–2010). However, in the statistical data, this ratio varies across climate zones. Regions with cold and temperate climate have low stocking density to grassland productivity ratios (0.75 ± 0.49 and 0.75 ± 0.48 LSU per 10 tonnes of DM, respectively), much less than that modeled by ORCHIDEE-GM. The highest ratio (1.49 ± 1.24 LSU per 10 tonnes of DM), close to the simulated one, is found in regions with a Mediterranean climate (Table 6).

**Table 6:** Ratio of livestock density to grassland productivity in Europe.

Köppen-Geiger climate zones	Livestock density / productivity LSU per 10 tonnes of DM	
	Model	Statistics
Clod climate	1.58±0.11	0.75±0.49
Temperate climate	1.72±0.15	0.75±0.48
Mediterranean climate	1.75±0.13	1.49±1.24

Note: See S2 Fig for map of Köppen-Geiger climate zones.

### Evaluation of grass-fed livestock numbers in Europe

**Fig 9:**
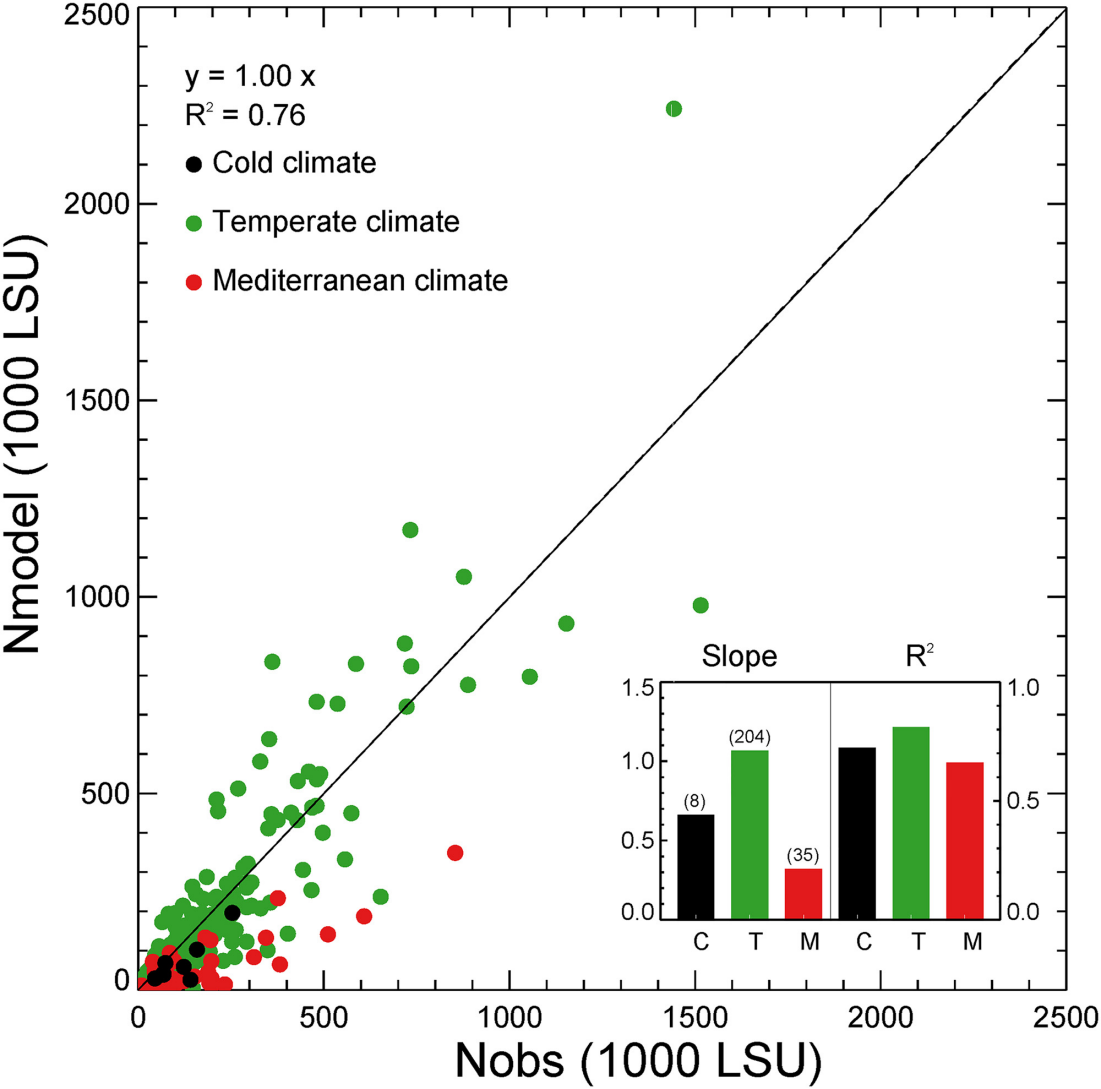
Comparison of modeled (*N*_*model*_) and ‘observed’ (*N*_*obs*_) grass-fed livestock numbers (‘observed’ numbers are inferred by combining Eurostat agricultural statistics and additional data in Eq 13) at the scale of each NUTS-2 region for the period 1990–2010 using ordinary least squares linear regression forced through the origin. One point is the average over a NUTS-2 region. A regression coefficient (*slope*) close to unity with a high coefficient of determination (R^2^) with P < 0.05 indicates a good model-data agreement. Climate zones follow the Köppen-Geiger classification (see text).

**Fig 10:**
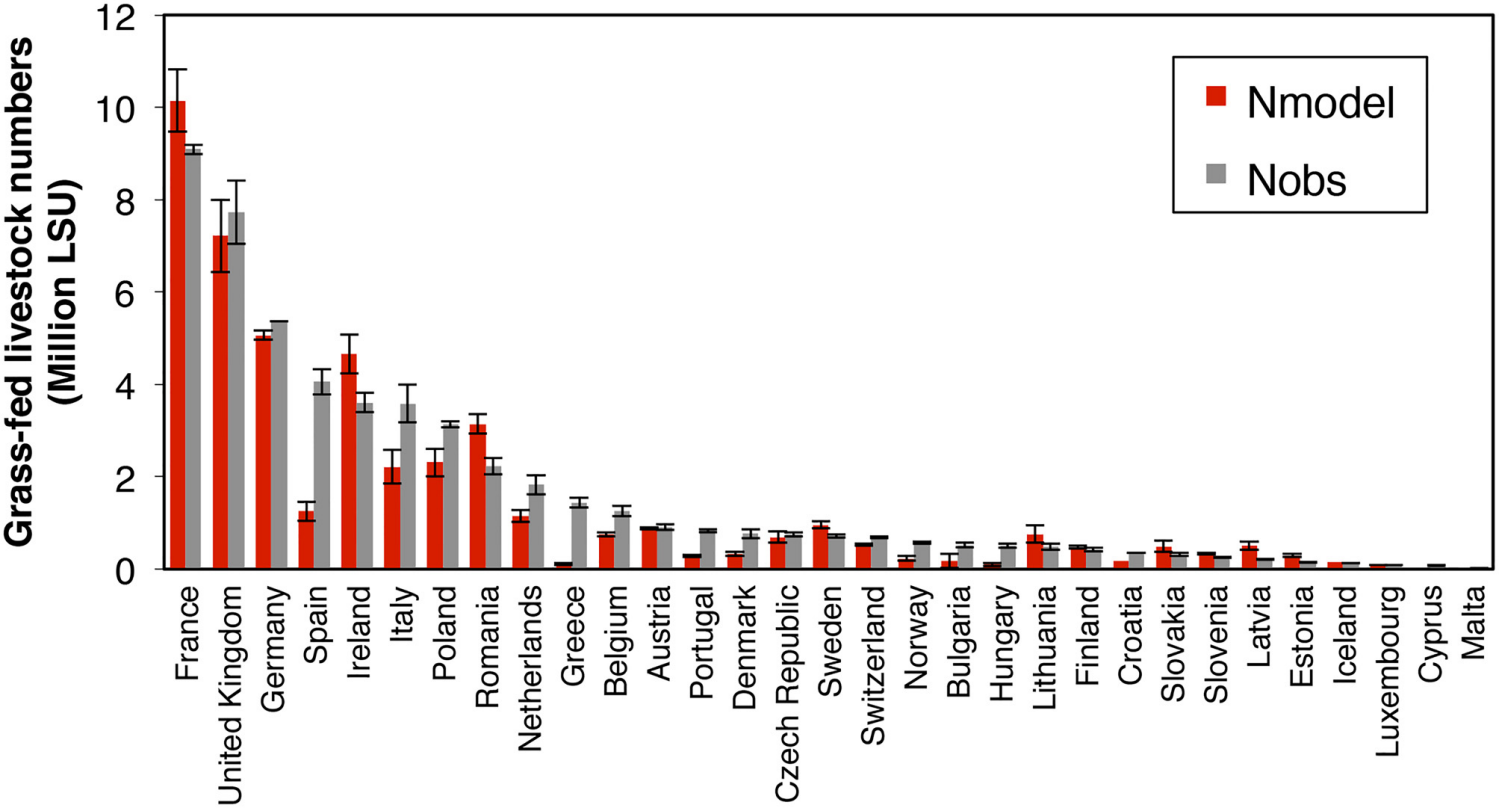
Comparison of modeled (*N*_*model*_) and ‘observed’ (*N*_*obs*_) (inferred from Eurostat and other data in Eq 13) grass-fed livestock numbers at the scale of NUTS-0 regions in Europe (countries) for the period 1990–2010. LSU: livestock unit.

Regressing the simulated numbers of grass-fed animals (*N*_*sim*_ in Eq 14) against the Eurostat data (*N*_*obs*_ in Eq 13), gives a (spatial) coefficient of determination R^2^ = 0.76 (P < 0.01) for the NUTS-2 regions (slope = 1.00; NUTS-1 for Germany; Fig 9). Country scale (NUTS-0) comparison (Fig 10) shows the mean value and standard deviation of the observed and modeled grass-fed livestock numbers during the period 1990–2010. When the comparison is made for regions grouped into Köppen-Geiger climate zones (S2 Fig; [77]), *N*_*sim*_ is comparable to *N*_*obs*_ in regions with temperate climate (slope close to 1, R^2^ > 0.8, P < 0.01; Fig 9). However, ORCHIDEE-GM tends to underestimate the number of grass-fed livestock in regions with cold high latitude climate (e.g., Norway) and in Mediterranean countries (e.g., Greece, Italy, Spain, Portugal).

## Discussion

### Spatial distribution of grassland productivity

In this study, the grassland productivity simulated by ORCHIDEE-GM represents a biological potential estimate (in equilibrium with climate and CO_2_ concentration), but neglects soil nutrient limitations on herbage production (not alleviated by nitrogen fertilization and deposition, a factor being accounted for in ORCHIDEE-GM simulations) and also ruminant nutrition limitations caused by low herbage quality. On the contrary, the statistical data compile actual production (often estimated from expert judgments) in each region under different types of management, which is often restricted by limiting soil nutrients and by low herbage quality. This difference is a major cause of productivity overestimation by the model. Another cause could be the possible overestimation of NPP by ORCHIDEE-GM (Table 3, see also [78]). NPP established from ecological measurements, separating aboveground from belowground productivity, is a more accurate indication of annual grass growth than grassland production estimates based on statistics. However, given the limited number of NPP measurements used to validate the model, its accuracy for grassland NPP projections cannot be fully ascertained.

Nevertheless, the spatial distribution of modeled potential productivity agrees well with statistics (Fig 4). ORCHIDEE-GM realistically reproduces (i) high productivity in humid regions, oceanic regions, and in wet mountain regions at low to mid-elevation, and (ii) low productivity in dry regions with a Mediterranean climate heavily affected by drought. This pattern confirms the major role played by water limitation in the productivity of temperate grassland ecosystems [79–84]. Then again, site-specific properties such as soil texture, terrain slope, nutrient availability, and grass species are not taken into account in the current model version, which reduce its ability to capture the actual details of the spatial distribution of grassland productivity. For example, we have a large overestimate of productivity in southern Europe (Fig 4) possibly due to the model’s lack of consideration of soil nutrients (S3A Fig) and to the low aboveground productivity of some grass species. On the other hand, overestimation of grassland productivity in southeast European countries (e.g., Romania and Bulgaria) may be the result of less grazing management reducing herbage productivity [7] and steppic conditions in some areas. Detailed spatial information about soil fertility and landscape management intensity would be helpful for improving the model capacity to simulate grassland productivity. On the other hand, some data limitations in terms of quality, transparency and detail [20] could be another cause of model-data discrepancies.

### Decadal trends and interannual variability of grassland productivity

For European grasslands, ORCHIDEE-GM estimates an average annual increase of potential productivity over the last five decades (0.36% ± 0.06% per year, Fig 7) which in the model is explained by the effects of elevated CO_2_, climate change, nitrogen fertilization, and nitrogen deposition. Changes in other management drivers (except nitrogen fertilization) in this period are not included in the model because the data (e.g., plant breeding) are not available. Meta-analyses revealed that nitrogen fertilization itself could stimulate grass biomass by around 50% [36, 37], and the positive biomass responses could be significantly enhanced when CO_2_ enrichment and/or the addition of other nutrients (such as phosphorus) were taken into account [36, 85]. Such a strong effect of fertilization, however, was not observed in regional-scale productivity statistics, nor in modeled potential productivity. We believe there are two reasons for this lack of response: i) Intensive fertilization that would strongly improve productivity has not been fully applied to all grasslands in Europe (a large part of the total grassland area was fertilized with only 0–40 kg N ha^-1^ yr^-1^, S1 Fig). ii) Fertilization and the improvement of grass species by plant breeding were introduced only a few decades ago in sown (i.e. temporary) grasslands, and with apparently limited effects on productivity. On the other hand, it has been shown that water stress is a major limitation on grassland productivity [79, 82, 83] and a small number of grasslands are irrigated in Europe [86]. Thus, in contrast to arable agriculture (annual crops), grasslands, which are still largely semi-natural in Europe, display changes in productivity over recent decades that are primarily controlled by climatic and atmospheric factors and not by management. Nevertheless, despite being increasingly decoupled from local grass productivity (e.g., increased use of crops and compound feeds in ruminant diet [2]), the livestock production (i.e., the potential grass-fed livestock density determined by potential productivity in this study) is still significantly influenced by trends in atmospheric CO_2_ concentration and climate (Table 4). The effect of these trends have to be taken into account for future scenarios, because a change of grassland productivity will imply significantly less of more feed complements that strongly related to agricultural policy.

Land-use change could be another significant and non-modeled factor that impacts long-term trends of grassland productivity in some EU countries. Transition to arable crops and abandonment can both mark a land-use change affecting grasslands. For example, in the Czech Republic, besides the impact of unfavorable climate factors captured by our model (Fig 8), grasslands were also abandoned during the transition to a market economy early 1990s, causing a drop in productivity between 1989 and 1992. In Italy, the evolution of grassland productivity has also been impacted by major land-use changes. The number of grazing animals decreased owing to the abandonment of low-productive dry rangelands [87]. At the same time, grassland areas with favorable conditions have been partly converted to fodder maize and to cash crops [87].

In certain regions, changes in the management of grasslands, such as fertilization, may have reduced the variability of productivity between years. However, significant interannual variability of grassland production was observed (Fig 8) in some countries because of climatic variability. In this study, we have shown that ORCHIDEE-GM is able of capturing the interannual variability of grassland productivity (Table 5).

### Grassland productivity uncertainties from model parameters

The key model inputs and parameters considered for uncertainty assessment in this study cause an uncertainty of potential grassland productivity (on average 1-sigma error of 0.8 tonnes DM ha^-1^ yr^-1^), and of its decadal trends (on average 1-sigma error of 0.06% per year), which is 19% of the average potential grassland productivity (4.4 tonnes DM ha^-1^ yr^-1^), and 17% of its average trend (0.36% per year). Similar magnitude of uncertainties, expressed in percentage of average value, was found on the grass-fed livestock density (19%) and the decadal trends of it (13%). Within the total uncertainty on the trends of potential productivity, the uncertainties caused by the parameters related to the response of grass photosynthesis to nitrogen addition effect (constant *a* and *Nadd*_*max*_) are small (contributing an uncertainty of 0.03% per year for each of them). It implies that the maximum response of photosynthetic capacity to nitrogen addition and the shape of saturating curve (Eq 3) have very limited effects on the trends of potential productivity estimate. The larger uncertainties come from parameters representing photosynthetic and morphological plant traits (*Vcmax*_*opt*_ */ Jmax*_*opt*_, and *SLA*_*max*_), which contribute a productivity uncertainty of 0.05% per year for each of them. The uncertain values of these parameters could be one of the sources for model-data disagreement when simulating C fluxes at sites [29]. However, these PFT-specific average plant functional traits in ORCHIDEE, in reality, are highly site-specific, but not with large variation on average. To narrow the uncertainty of the traits-related parameters, improved observation datasets on both mean value at community level (contrary to species level) and on spatial distribution are required. Meanwhile, these traits are tightly correlated with leaf nitrogen concentrations [88] suggesting a possible way to reduce the uncertainty by fully coupling nitrogen and carbon cycles in terrestrial ecosystems [39].

### Livestock density and its ratio with grassland productivity

Spatial distribution of grass-fed livestock density estimated by ORCHIDEE-GM is in agreement with the general pattern of actual ruminant livestock distribution given by FAO [62] (Fig 5). However, because FAO data are densities expressed as livestock per unit of land area suitable for livestock production, data from regions with reduced grassland cover, or with low intensity of grassland utilization by livestock, should not be compared with ORCHIDEE-GM output. The actual livestock density is also restricted by soil quality, terrain slope, and by socioeconomic factors. Regions with low livestock density (< 0.2 LSU ha^-1^) are distributed across areas with high levels of soil workability constraints (i.e., soil conditions to ploughing, level 2–4 in S3A Fig; moderate constraints or (very) severe constraints) and high terrain slope (> 16% in S3B Fig), including Norway, Sweden, Scotland, Mediterranean regions, and mountainous areas. Those soil and terrain conditions prevent the use of mechanized mowing and, hence, the harvesting of large stocks of conserved forage, which is a prerequisite if animals are to be stocked at a high density during the grassland growing season. Moreover, the low livestock density in eastern Europe may be restricted by socioeconomic conditions as well.

The model simulations of the ratio of livestock density to grass production are derived under the assumption of maximum densities supported by the productivity of each grid cell and the maximum utilization of the grassland productivity by grazing and mowing. This assumption implies a fully utilized biological potential. When derived from statistics this ratio is an indicator of the grassland utilization intensity. The ratio from statistics in Mediterranean regions is close to that derived from model simulations, indicating an intensive use, or even an over-utilization (i.e., overgrazing), of grasslands. The low value of the statistical ratio in temperate Europe (especially in eastern Europe; data not shown) is related to an extensive use of grasslands, resulting from unfavorable conditions in mountainous areas (Slovakia, Romania and Bulgaria; S3B Fig), to the widespread abandonment of grassland areas in former communist countries [89], and to non-intensive management.

### Regional numbers of grass-fed livestock

Grass-fed livestock numbers simulated by ORCHIDEE-GM are comparable with the data inferred from Eurostat and FAO statistics, yet with some discrepancies (Figs 9 and 10). Model-data misfits in the numbers of grass-fed livestock units could be caused by: i) the assumption used in Eqs 4 and 5 that grasslands are either cut or grazed, whereas a number of grassland areas in Europe have a mixed use (e.g., combining a spring or summer cut and several grazing periods per year [7, 75], ii) the fact that cut grasslands sustain more head of livestock per hectare than grazed grasslands (since the fraction of the total aboveground dry matter harvested by mowing is greater than with grazing; see Fig 3 and [32]). The proportion of cut grasslands (*1-F*_*opt*_, in Eq 4) in the real world could be higher than our optimal value in some regions (e.g., Denmark, northern Germany and Austria [90]), given the prevalence in these countries of zero-grazing for dairy cattle. If so, grasslands in these regions are able to feed more livestock than the numbers estimated by the model. iii) Animal diet composition in each region is different because of differences in the ratio of cropland to grassland area, in feed imports and exports, and in socioeconomic conditions. Thus, the coarse estimate of the fractional component of grass in animal diet used in our model-data comparison (only aggregated data for western Europe and eastern Europe were available in [3], the only data source about the grass-fed fraction of animals to the best of our knowledge) could be a source of inaccuracy in estimating grass-fed livestock numbers. iv) The factors limiting the utilization intensity for each kind of grassland (*f*_*temp*_, *f*_*perm*_ and *f*_*rough*_ in Eq 14) are assumed fixed over all of Europe. However, in reality these parameters may be different for different regions [87]. For example, in the continental, boreal or Mediterranean zones, permanent grasslands tend not to be plowed and are less intensively managed, thus with an *f*_*perm*_ lower than the default value (80%), which may induce a model overestimation of livestock density in these regions. v) In Mediterranean zones, where livestock is mostly sheep and goats, animals sometimes graze in shrubland or forest [91–93], which is not considered in our model. For the Mediterranean zones, the tendency of ORCHIDEE-GM to underestimate grass-fed livestock numbers (Fig 9) while overestimating productivity (Fig 4), could be partly explained by the particular farm structure (with sheep and goats as the major ruminant type [62]) and by overgrazing in some regions (e.g. [94]). Meanwhile, this result suggests that integrated productivity data (i.e., data in [20]) in Mediterranean zones might underestimate grassland productivity.

Accuracy of data is another important factor inducing discrepancies with model results: i) For some countries, grassland areas from FAOstat [5] and Eurostat [41] are significantly different (S2 Table). For example, grassland area from FAOstat [5] is larger than that from Eurostat [41] for permanent grassland in Bulgaria (by 39%), Greece (by 499%), Hungary (by 39%), Italy (by 29%), Spain (by 25%), Switzerland (by 77%) and United Kingdom (by 16%), and for temporary grassland in Germany (by 440%) and Spain (by 94%). This may partly explain the underestimation of grass-fed livestock numbers in these countries, because in this study grassland area was taken from Eurostat [41] (Eq 14). ii) For some countries, the area of grassland is very different from year to year. In Bulgaria (S3 Table) for instance, the areas of grasslands change between each survey, which may be due to changes in the criteria used for the surveys.

## Conclusion and Outlook

This study provides an estimation of potential productivity and potential livestock density over European grasslands by using the process-based ecosystem global vegetation model ORCHIDEE-GM with a representation of grassland management. When compared to statistical data from Eurostat or FAOstat, ORCHIDEE-GM can distinguish the spatial distribution of regions with low or high productivity and livestock density. However, the model tends to overestimate absolute values of productivity and livestock density because it was built and run to reproduce a biological potential. The model-data discrepancies reflect site specific properties such as soil quality, terrain slope, nutrient availability, grass species, management intensity, land-use change and socioeconomic factors that constrain productivity to be below the biological potential. Including these limitations in the model would probably allow future studies to estimate productivity more accurately.

The effects of elevated CO_2_, climate change, nitrogen fertilization and nitrogen deposition are well reflected in the temporal evolution of productivity simulated by the model. But at regional scale the evolution of managed grassland productivity is also determined by land-use change, and by land management intensity changes, both reflecting agricultural policy and socio-economical drivers. ORCHIDEE-GM is able to reproduce the interannual variability of productivity in three of the four countries where long-term data are available (the exception is Italy), making it likely that the model will be able to give useful predictions of the dynamics of potential productivity of European grasslands under future climate change.

In addition, grass-fed livestock numbers can be realistically reproduced by ORCHIDEE-GM using simple equations and parameterization. Regional model-data misfits exist due to uncertainties caused by different farming strategies (e.g., mowing or grazing, type of animal species), diet composition, management intensity, as well as the uncertainties in the measured data. ORCHIDEE-GM, with its capability to account for grassland management and livestock fed by grass, could contribute to our understanding of future changes in the European livestock sector and of climate impacts on grassland, as well as atmospheric feedbacks through GHG emissions and soil carbon balance.

## Supporting Information

S1 File Grids selection for productivity uncertainty analysis. (DOCX)

S1 Fig Spatial distribution of the amount of nitrogen addition (including both mineral and organic nitrogen fertilizer) over European grasslands for the year 2010. The grey areas in EU27 states indicate that nitrogen addition was not applied. The blank grids for Croatia, Norway, and Switzerland indicate that the data were not available.
(TIFF)

S2 Fig Köppen-Geiger climate classification map of Europe adapted from Peel et al. [77]. Cold climate, also as tundra climate (ET); Temperate climate, including moist mid-latitude climate with cold winters (Df), and with mild winters (Cf); Mediterranean climate (Cs). ET, Df, Cf, and Cs are original climate types used in Peel et al. [77].
(TIFF)

S3 Fig (A): The soil workability constraints, and (B): the median terrain slope class from the Global Agro-Ecological Zones assessment (GAEZ, http://www.fao.org/nr/gaez/en/). The soil workability constraints are classified into four levels: 1, no or slight constraints; 2, moderate constraints; 3, severe constraints; and 4 very severe constraints.
(TIFF)

S1 Table Fraction of grass fed in ruminant livestock diet (%, dry matter basis) [3]. (DOCX)

S2 Table Grassland area of European countries from FAO [5] and Eurostat [41] (Unit: 1000 hectare). (DOCX)

S3 Table Area of grassland of Bulgaria from Eurostat [39] (unit: hectare). The year indicates the year of farm surveys.
(DOCX)
