# Supplementary material for: Modeled Changes in Potential Grassland Productivity and in Grass-Fed Ruminant Livestock Density in Europe over 1961–2010
Source: PLoS One. 2015 May 27;10(5):e0127554. doi: 10.1371/journal.pone.0127554 (PMC4446363; doi:10.1371/journal.pone.0127554)
Supplement: S1 File — (DOCX) [file pone.0127554.s001.docx]

S1 File. **Grids selection for productivity uncertainty analysis**

Complete simulations (as described in section “Simulation set-up”), including a 10000 years spin-up simulation to reach the equilibrium of carbon pools and a 110 years simulation for the period of 1901 to 2010, with the 16 factors combinations at full geographical scale (9237 grids) require enormous computational time which is beyond our capability. Thus for the uncertainty analysis in this study, we can only make the series of simulations at a reasonable number of grids with affordable computational demand. Importantly, the grids should be able to represent the spatial distribution, magnitude and interannual variability of potential grassland productivity. We carried out a series of selection processes which picked grids with regular latitude / longitude intervals (from 0.25° to 3°, which is 1 to 12 folds of the spatial resolution in this study). As a result, 12 groups of grids that regularly spread in the study area were formed with 58 to 2309 grids over the study area (Table A1; Figure A1 as an example). For each group, the potential grassland productivity of the corresponding grids were extracted and averaged, then were compared with that at full geographical scale of 9237 grids (control group). Figure A2 shows the differences in average productivity (with respect to magnitude of productivity) and the correlation coefficient between productivity time series from all grids (control group) and from each group of grids (with respect to interannual variability of productivity). Finally, group 4 (368 grids) was selected for the uncertainty analysis in section “Uncertainties on the potential grassland productivity and grass-fed livestock density estimation”, given the fact that its average productivity is close to that of control group and relatively high correlation coefficient (Figure A2).

Table A Grids chosen by the selection processes with different latitude / longitude intervals

| Group | Latitude/Longitude interval (°) | Number of grids |
| --- | --- | --- |
| Control | 0 | 9237 |
| G1 | 0.25 | 2309 |
| G2 | 0.5 | 1017 |
| G3 | 0.75 | 583 |
| G4 | 1 | 368 |
| G5 | 1.25 | 244 |
| G6 | 1.5 | 195 |
| G7 | 1.75 | 145 |
| G8 | 2 | 114 |
| G9 | 2.25 | 92 |
| G10 | 2.5 | 76 |
| G11 | 2.75 | 62 |
| G12 | 3 | 58 |


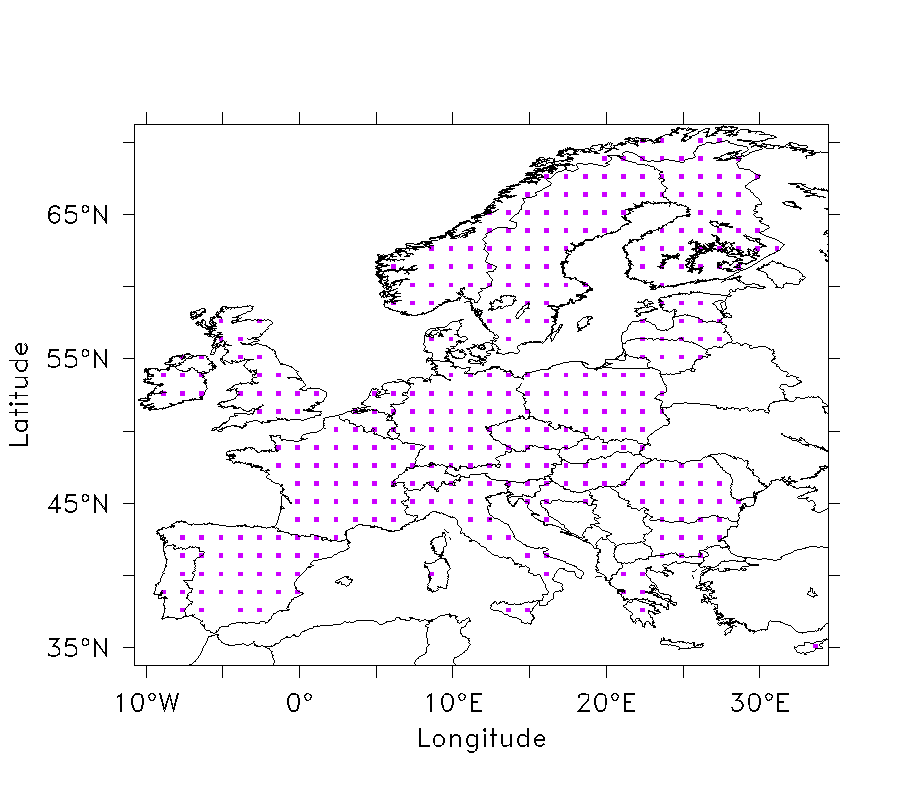


Figure A The spatial distribution of the selected grids. Figure A1 exemplifies the grids of G4 (with latitude / longitude interval of 1°).


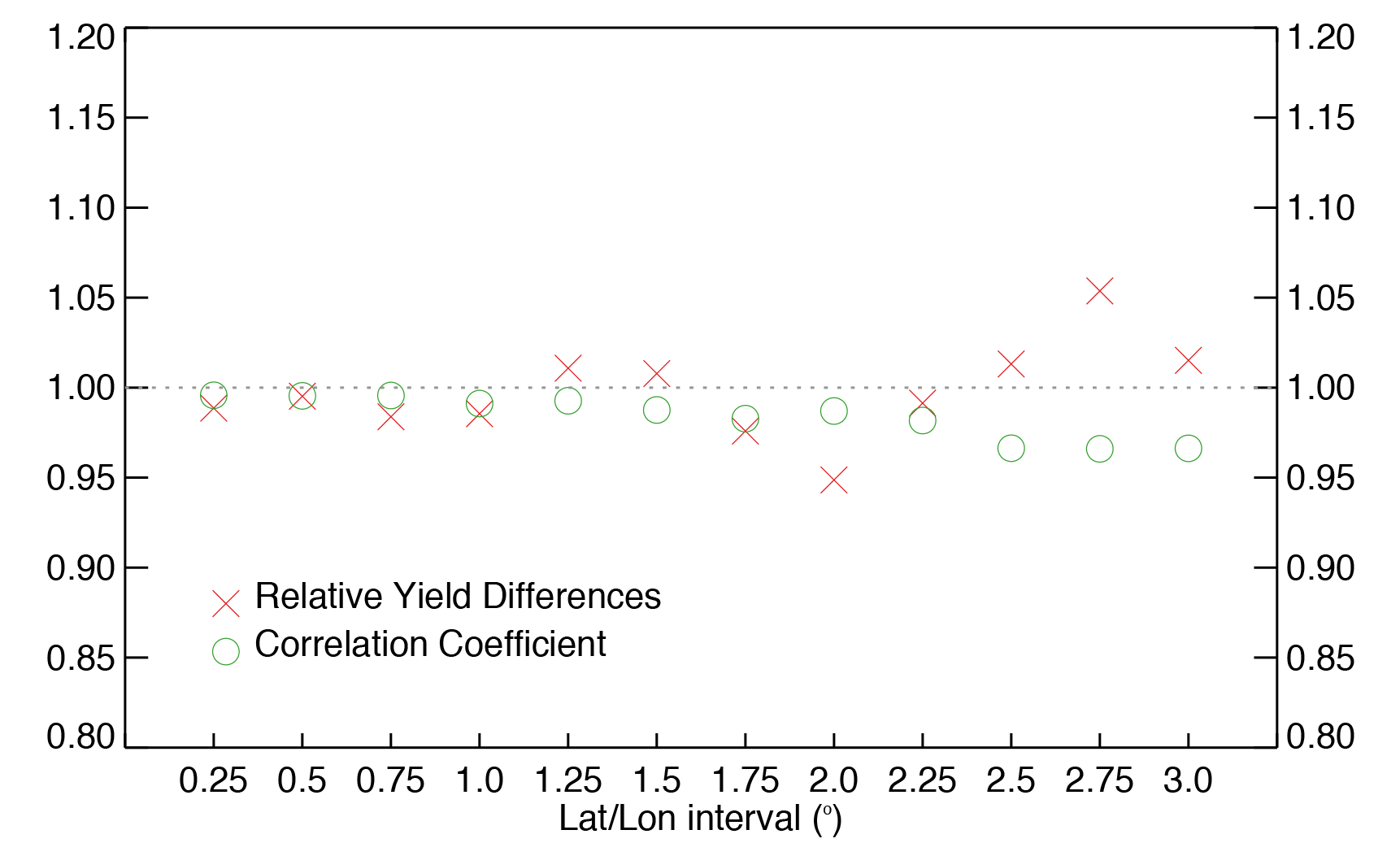


Figure B The differences in average productivity (*yield*) and the correlation coefficient between productivity time series from all grids (control group) and from each group of grids. The differences in average productivity were expressed as the ratio of average productivity of each group to average productivity of control group.
