# Supplementary figures and images for: Modeled Changes in Potential Grassland Productivity and in Grass-Fed Ruminant Livestock Density in Europe over 1961–2010
Source: PLoS One. 2015 May 27;10(5):e0127554. doi: 10.1371/journal.pone.0127554 (PMC4446363; doi:10.1371/journal.pone.0127554)

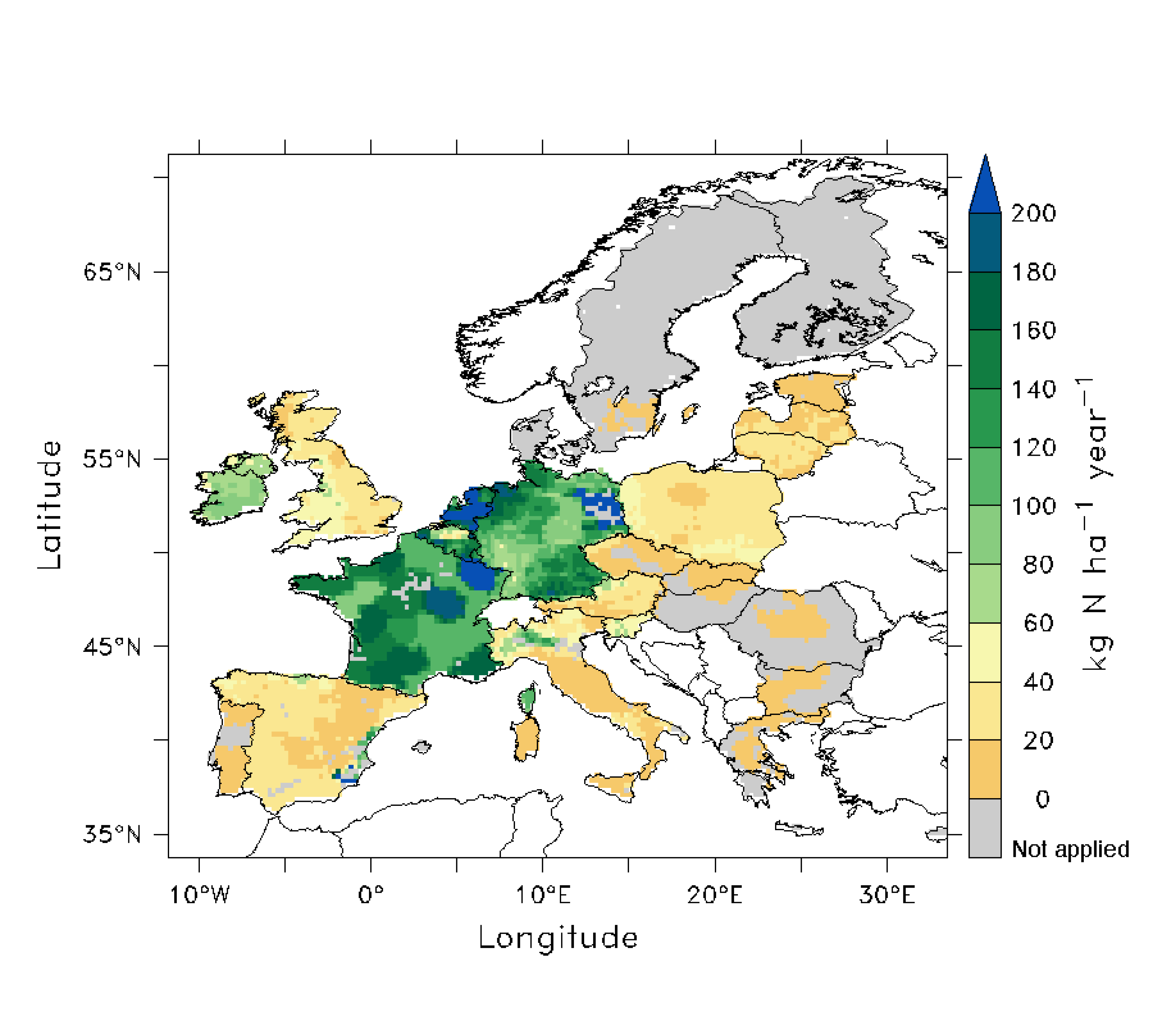

Supplement: S1 Fig — The grey areas in EU27 states indicate that nitrogen addition was not applied. The blank grids for Croatia, Norway, and Switzerland indicate that the data were not available. (TIFF) [file pone.0127554.s002.tiff]

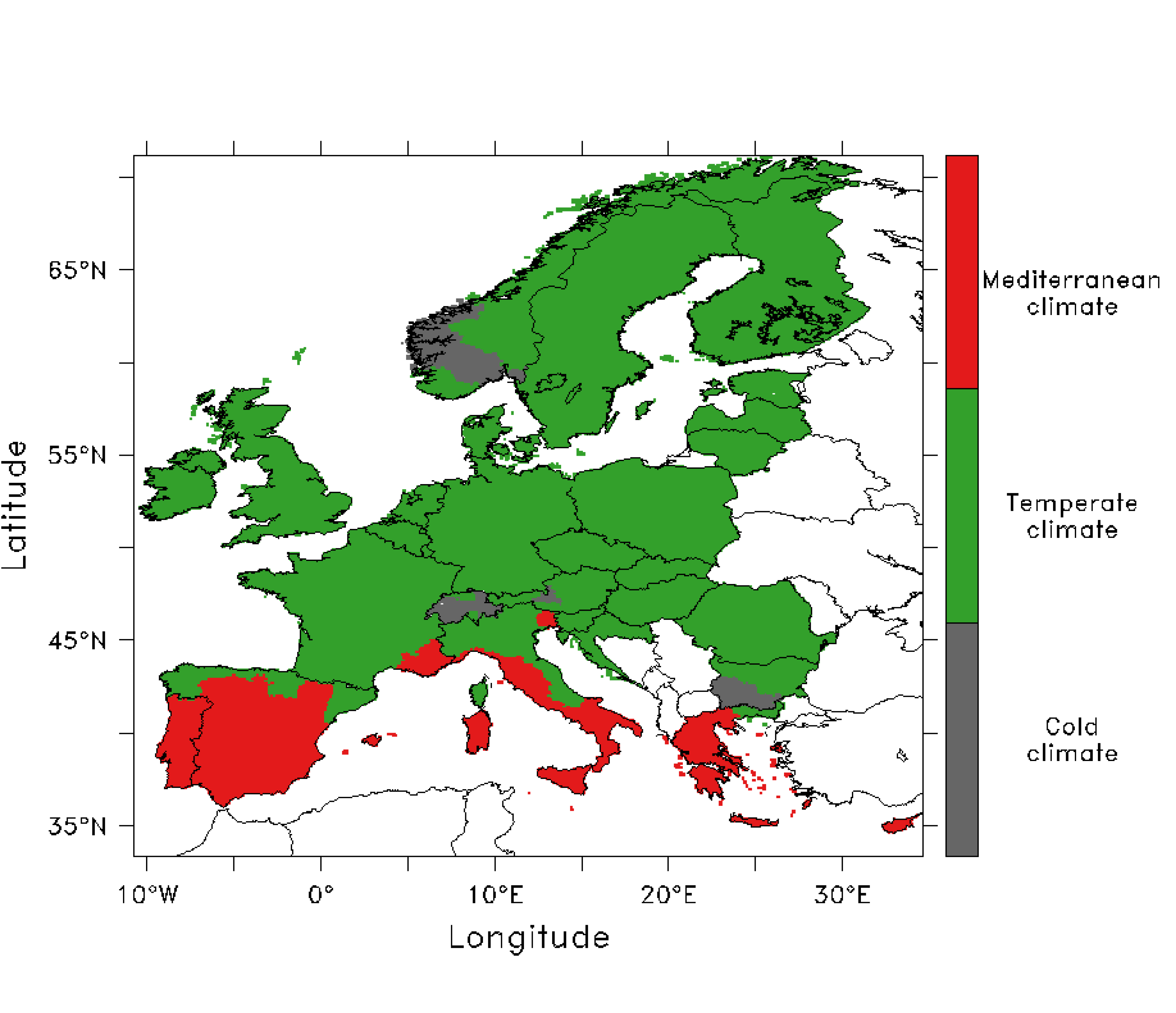

Supplement: S2 Fig — Cold climate, also as tundra climate (ET); Temperate climate, including moist mid-latitude climate with cold winters (Df), and with mild winters (Cf); Mediterranean climate (Cs). ET, Df, Cf, and Cs are original climate types used in Peel et al. [77]. (TIFF) [file pone.0127554.s003.tiff]

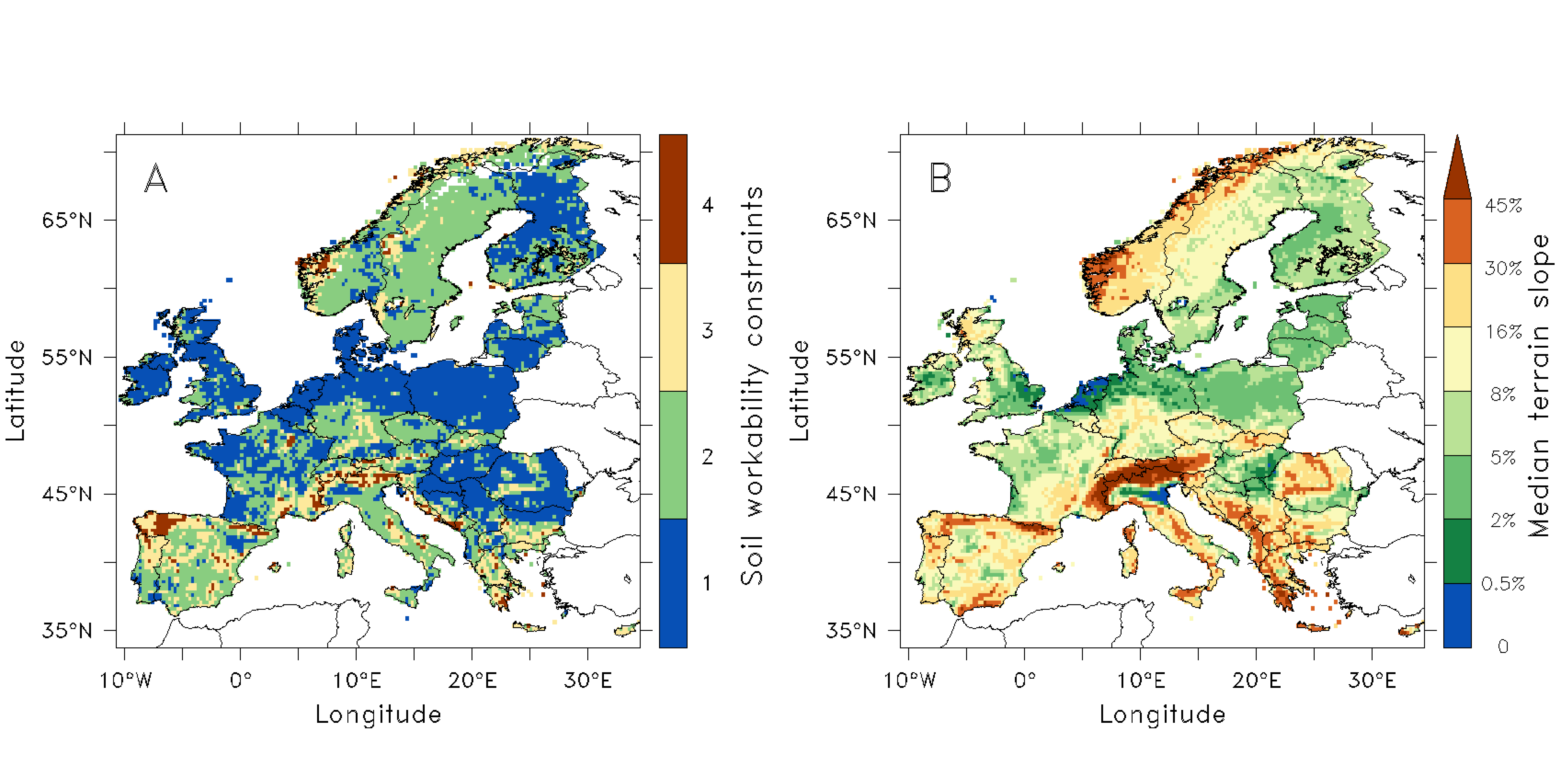

Supplement: S3 Fig — The soil workability constraints are classified into four levels: 1, no or slight constraints; 2, moderate constraints; 3, severe constraints; and 4 very severe constraints. (TIFF) [file pone.0127554.s004.tiff]
