# Supplementary material for: Modeled Changes in Potential Grassland Productivity and in Grass-Fed Ruminant Livestock Density in Europe over 1961–2010
Source: PLoS One. 2015 May 27;10(5):e0127554. doi: 10.1371/journal.pone.0127554 (PMC4446363; doi:10.1371/journal.pone.0127554)
Supplement: S1 Table — (DOCX) [file pone.0127554.s005.docx]

S1 Table. Fraction of grass fed in ruminant livestock diet (%, dry matter basis) [3]

|  | Beef cattle | | Dairy cattle | | Sheep and Goats | |
| --- | --- | --- | --- | --- | --- | --- |
|  | 1993 | 2030 | 1993 | 2030 | 1993 | 2030 |
| Eastern Europe | 71.1 | 73.7 | 70.2 | 68.4 | 81.5 | 85.2 |
| Western Europe | 64.1 | 64.6 | 49.1 | 44.4 | 77.5 | 85.9 |
