# Supplementary material for: Modeled Changes in Potential Grassland Productivity and in Grass-Fed Ruminant Livestock Density in Europe over 1961–2010
Source: PLoS One. 2015 May 27;10(5):e0127554. doi: 10.1371/journal.pone.0127554 (PMC4446363; doi:10.1371/journal.pone.0127554)
Supplement: S2 Table — (DOCX) [file pone.0127554.s006.docx]

S2 Table. Grassland area of European countries from FAO [5] and Eurostat [39] (Unit: 1000 hectare).

| Country | Permanent grassland | | Temporary grassland | |
| --- | --- | --- | --- | --- |
|  | FAO | Eurostat | FAO | Eurostat |
| Austria | 1731 | 1439 | 66 | 60 |
| Belgium | 502 | 500 | 81 | 79 |
| Bulgaria | 1719 | 1241 | 76 | 1 |
| Cyprus | 4 | 2 |  | 0 |
| Czech Republic | 983 | 929 |  | 36 |
| Denmark | 197 | 200 | 492 | 321 |
| Estonia | 327 | 296 | 170 | 124 |
| Finland | 34 | 33 | 639 | 649 |
| France | 9870 | 8419 | 3130 | 3198 |
| Germany | 4741 | 4655 | 2091 | 387 |
| Greece | 4500 | 751 | 229 | 118 |
| Hungary | 1004 | 721 | 13 | 18 |
| Ireland | 3097 | 3979 | 724 | 659 |
| Italy | 4423 | 3434 |  | 1082 |
| Latvia | 659 | 651 |  | 326 |
| Lithuania | 608 | 606 | 412 | 541 |
| Luxembourg | 67 | 68 |  | 11 |
| Netherlands | 827 | 813 | 197 | 182 |
| Norway | 175 | 362 | 487 | 477 |
| Poland | 3180 | 3229 |  | 124 |
| Portugal | 1781 | 1785 |  | 31 |
| Romania | 4372 | 4506 | 819 | 90 |
| Slovakia | 524 | 531 | 47 | 71 |
| Slovenia | 267 | 286 | 23 | 24 |
| Spain | 10464 | 8377 | 1021 | 329 |
| Sweden | 436 | 452 |  | 1148 |
| Switzerland | 1095 | 620 | 125 | 131 |
| United Kingdom | 11233 | 9705 | 1178 | 1219 |
