# Supplementary material for: Modeled Changes in Potential Grassland Productivity and in Grass-Fed Ruminant Livestock Density in Europe over 1961–2010
Source: PLoS One. 2015 May 27;10(5):e0127554. doi: 10.1371/journal.pone.0127554 (PMC4446363; doi:10.1371/journal.pone.0127554)
Supplement: S3 Table — The year indicates the year of farm surveys. (DOCX) [file pone.0127554.s007.docx]

S3 Table. Area of grassland of Bulgaria from Eurostat [39] (unit: hectare). The year indicates the year of farm surveys.

| Type of grassland | 2003 | 2005 | 2007 | 2010 |
| --- | --- | --- | --- | --- |
| Permanent Pasture and Meadow | 95,680 | 73,980 | 166,460 | 628,070 |
| Rough grazing | 11,710 | 32,940 | 82,140 | 556,460 |
| Temporary grassland | 75,100 | 58,490 | 68,540 | 1090 |
